# Application of chloroplast genome in the identification of Traditional Chinese Medicine *Viola philippica*

**DOI:** 10.1186/s12864-022-08727-x

**Published:** 2022-07-27

**Authors:** Dong-Ling Cao, Xue-Jie Zhang, Shao-Qiu Xie, Shou-Jin Fan, Xiao-Jian Qu

**Affiliations:** Shandong Provincial Key Laboratory of Plant Stress Research, College of Life Sciences, Shandong Normal University, Ji’nan, 250014 China

**Keywords:** *Viola philippica*, Identification, Chloroplast genome, Unique variable site, Morphological characteristics, Phylogeny

## Abstract

**Background:**

*Viola philippica* Cav. is the only source plant of “Zi Hua Di Ding”, which is a Traditional Chinese Medicine (TCM) that is utilized as an antifebrile and detoxicant agent for the treatment of acute pyogenic infections. Historically, many *Viola* species with violet flowers have been misused in “Zi Hua Di Ding”. *Viola* have been recognized as a taxonomically difficult genera due to their highly similar morphological characteristics. Here, all common *V. philippica* adulterants were sampled. A total of 24 complete chloroplast (cp) genomes were analyzed, among these 5 cp genome sequences were downloaded from GenBank and 19 cp genomes, including 2 “Zi Hua Di Ding” purchased from a local TCM pharmacy, were newly sequenced.

**Results:**

The *Viola* cp genomes ranged from 156,483 bp to 158,940 bp in length. A total of 110 unique genes were annotated, including 76 protein-coding genes, 30 tRNAs, and four rRNAs. Sequence divergence analysis screening identified 16 highly diverged sequences; these could be used as markers for the identification of *Viola* species. The morphological, maximum likelihood and Bayesian inference trees of whole cp genome sequences and highly diverged sequences were divided into five monophyletic clades. The species in each of the five clades were identical in their positions within the morphological and cp genome tree. The shared morphological characters belonging to each clade was summarized. Interestingly, unique variable sites were found in *ndhF*, *rpl22*, and *ycf1* of *V. philippica*, and these sites can be selected to distinguish *V*. *philippica* from samples all other *Viola* species, including its most closely related species. In addition, important morphological characteristics were proposed to assist the identification of *V. philippica*. We applied these methods to examine 2 “Zi Hua Di Ding” randomly purchased from the local TCM pharmacy, and this analysis revealed that the morphological and molecular characteristics were valid for the identification of *V. philippica*.

**Conclusions:**

This study provides invaluable data for the improvement of species identification and germplasm of *V. philippica* that may facilitate the application of a super-barcode in TCM identification and enable future studies on phylogenetic evolution and safe medical applications.

**Supplementary Information:**

The online version contains supplementary material available at 10.1186/s12864-022-08727-x.

## Background

As the largest genus of Violaceae, *Viola* is mainly distributed in temperate and tropical regions, and China is one of the distribution centers [1–3]. More than 20 *Viola* species have been recorded as medicinal plants with definite efficacy and indications [4–7]. *V. philippica* (Herba Violae) possesses significant and unique efficacy in clinical antiviral therapy. Research has shown that *V. philippica* extract possess a wide range of pharmacological and biological activities, including antiviral, antifungal, anticoagulant and anticancer functions [8, 9]. Notably, cyclotides from *V. philippica* extracts are remarkably stable and tolerate harsh thermal, chemical, and enzymatic conditions with high biological activity, including insecticidal, cytotoxic, and neurotensin antagonistic activities. These characteristics make *V. philippica* ideal for potential agrochemical or pharmaceutical applications [10–12]. *V. philippica* is a small perennial herb with violet flowers. The dried whole plant (including the roots) is an important TCM named “Zi Hua Di Ding” in Chinese [13, 14]. “Zi Hua Di Ding” was the only *Viola* species described in the Chinese Pharmacopoeia in 1977, in which *V. philippica* is the only source of “Zi Hua Di Ding”; it has been widely used ever since [15]. Furthermore, the anti-HIV activity of *V. philippica* as traditional medicine was described in the 1989 World Health Organization (WHO) bulletin [16, 17]. Importantly, recent studies have demonstrated that its extracts exhibit high inhibitory activity against HIV-1 and respiratory syncytial virus (RSV) in vitro, with potential for efficacious clinical applications [18–21].

*Viola* is traditionally considered to be a morphologically difficult genus to classify [1, 2, 22]. Due to its wide distribution, frequent hybridization, and both open and closed flowers (both sexual and asexual), the morphological features of the *Viola* species exhibit high variability [23–26]. The infrageneric and interspecific relationships are confused due to similar morphological characteristics. The phylogenetic positions of some *Viola* species are difficult to identify. Unclear phylogenetic relationships and highly similar morphological characteristics among *Viola* species restrict the breeding and development of *V. philippica* germplasm resources. One or a more chloroplast DNA molecular markers, the nuclear intergenic transcribed spacer (ITS), or the inter-simple sequence repeat (ISSR) have previously been used to infer the phylogenetic relationships within *Viola* [27–29]. However, the interspecies phylogenetic relationships of *Viola* are still controversial due to insufficient informative sites and incomplete taxon sampling. Most prior studies have focused on the *Viola* species of North America, Korea, and Japan, while there are limited studies regarding *Viola* species from China. Flora of China proposed that the *Viola* species in China are divided into 14 sections based on morphological characteristics, including sections *Adnatae*, *Bilobatae*, *Brevicalcaratae*, *Caudicaules*, *Diffusae*, *Erectae*, *Longicalcaratae*, *Noverculae*, *Pinnatae*, *Plagiostigma*, *Serpentes*, *Trigonocarpae*, *Vaginatae*, and *Viola* [3, 30]. The range of sections and their identification depends only on morphological characteristics that are often disputed by taxonomists. Taxonomic and phylogenetic analyses of *Viola* based on molecular inference are frequently inconsistent with the conclusions of traditional views. The delimitation of sect. *Adnatae* and the phylogenetic position of the species within it are widely disputed. *V. philippica* belongs to the sect. *Adnatae*, which is a group of about 35 species in China. It is very difficult to accurately identify *V. philippica* for use in TCM by morphological characteristics or organoleptic methods. Ensuring the authenticity of source plants is a key issue in the use of herbs. Some species have been misidentified due to their violet-colored flowers being the same color as those of *V. philippica*, e.g., *Corydalis bungeana* or *Gueldenstaedtia verna*. Common adulterants in “Zi Hua Di Ding” are often *Viola* species, i.e., *V*. *patrinii*, *V*. *inconspicua*, *V*. *yezoensis*, *V*. *phalacrocarpa*, and *V*. *prionantha.* In practice, some micromorphological features are difficult to identify, especially those concentrated in the floral organs. The result is that many *Viola* species with violet flowers were often treated as *V. philippica*. Folk lacks botanical knowledge, confusing the safety and effectiveness of drug use. Consumers are often unable to verify original voucher specimens of herbs when they purchase them in pharmacies. Therefore, accurate and efficient identification of the source plants of “Zi Hua Di Ding” is necessary.

DNA barcoding is an emerging technology of molecular identification and classification; it significantly enhances the safety and efficacy of medicinal herbs [31–33]. DNA barcoding is not restricted by morphological characteristics or physiological conditions, allowing for species authentication without professional taxonomic knowledge [34, 35]. Chloroplast, the vital photosynthesis tissue, is an important organelle in green plants. The plastomic sequence is commonly described as a quadripartite structure with a large single-copy (LSC), a small single-copy (SSC), and a pair of inverted-repeat (IR) regions [36]. The angiosperm cp genomes are generally of moderate size, ranging from 120 to 160 kb [37–39]. With the rapid development of high throughput sequencing technology, the cp genome (chloroplast genome) is widely applied as a super-barcode, which could provide effective information for resolving phylogenetic relationships and identification of medicinal plants [40–42]. In the present study, we set out to analyze the cp genome to authenticate *Viola* species, in particular *V. philippica*. Furthermore, molecular markers in highly divergent cp genome regions were screened for the identification of *Viola* and phylogenetic studies.

Herein, a total of 22 *Viola* samples were obtained, for which cp genome sequences of three species were downloaded from GenBank and the cp genomes of 19 *Viola* samples were newly sequenced. The common adulterants in “Zi Hua Di Ding” belonging to the *Viola* were covered. The purpose of this study was to 1) compare the chloroplast genome structures of the sampled *Viola* species; 2) find an effective means to by which to distinguish *V. philippica* from other *Viola* species; and 3) resolve the infrageneric relationships within *Viola* species, especially section *Adnetae*, *Pinnatae*, and *Bilobatae*, using complete plastomic sequences and highly diverged sequences. This study provides invaluable data for species identification and improvement in germplasm generation of *V. philippica*; it will facilitate the application of super-barcode cp genomics in TCM identification, allowing for future studies on phylogenetic evolution and safe medical applications of *Viola* species.

## Results

### Cp genome organization of *Viola*

**Fig. 1:**
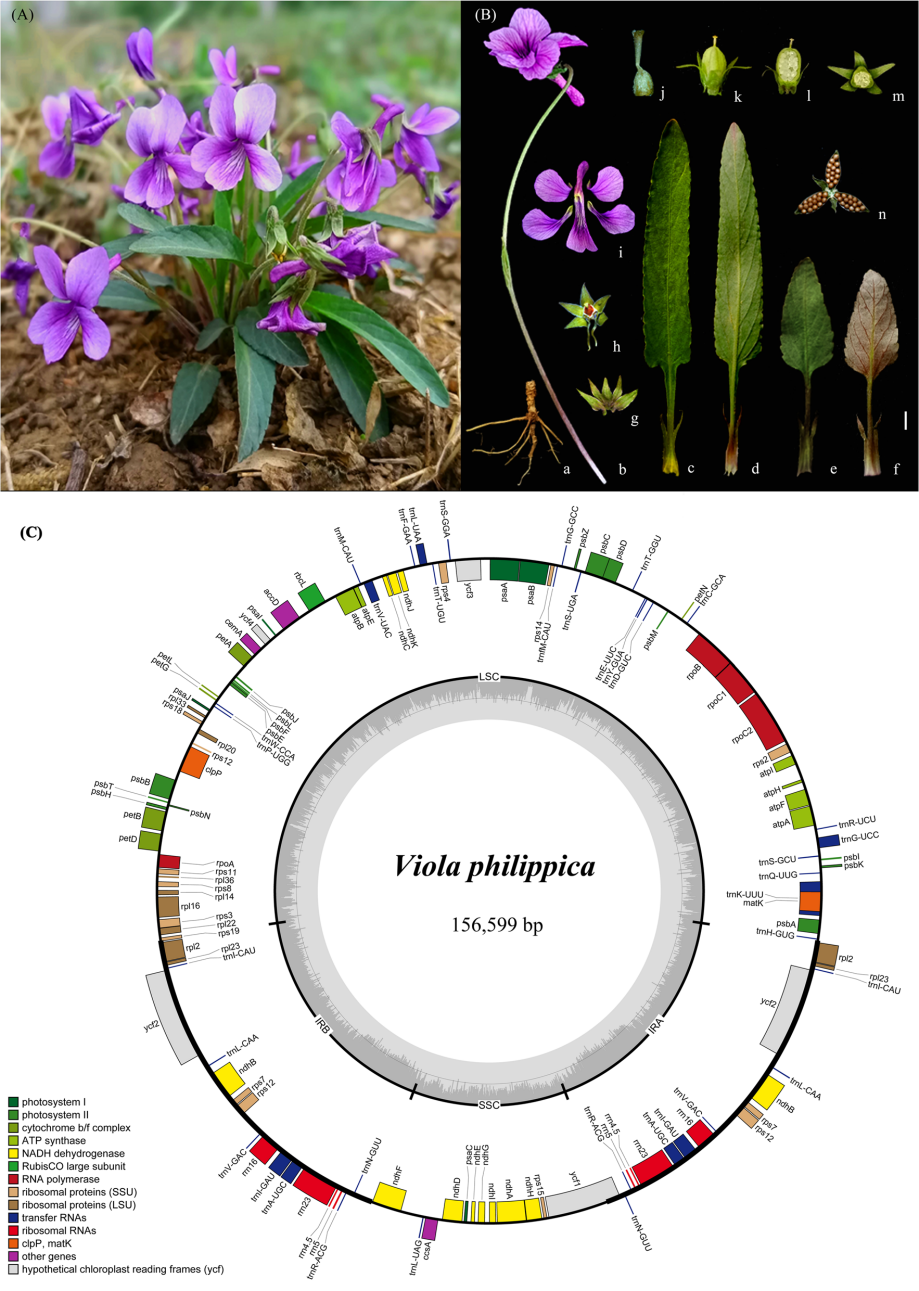
Morphology and cp genome map of *Viola* species. Photo credits: Dong-Ling Cao. **A** The morphological character of *V. philippica*. **B** a. root; b. flower; c. upper leaf blade (ventral); d. upper leaf blade (dorsal); e. lower leaf blade (ventral); f. lower leaf blade (dorsal); g. sepal; h. nectar glands; i. petal; j. style; k. capsule; l. capsule longitudinal section; m. capsule transverse section; n. capsule dehiscence. Scale bar =1cm in a; 50mm in b, c, d, e, f, g, h, i, and n; 5mm in k, l, m; 1mm in j. **C** Chloroplast genome map of *V. philippica* with annotated genes. The genes transcribed clockwise are shown inside of the circle whereas genes transcribed anti-clockwise are shown outside of the circle. The colored bars indicate different functional groups. The darker gray area in the inner circle denotes GC content while the lighter gray corresponds to the AT content of the genome. LSC: large single copy, SSC: small single copy, IR: inverted repeat

All *Viola* species analyzed in the current study that have been accurately identified possessed a similar genome structure, gene order, and orientation. Plastome size ranged from 156,483 bp (*V. phalacrocarpa*) to 158,940 bp (*V. acuminata*) (Table 1). The two *V. philippica* plastomes, approximately at 157 kb (Fig. 1). The 17 *Viola* species cp genomes had highly conserved quadripartite structure with the LSC region (85,364-87,250 bp), SSC region (16,558-18,008 bp), and a pair of IRs (26,404-27,404 bp). The overall guanine-cytosine (GC) content was approximately 36%. The GC contents in the LSC and SSC regions of all 17 species were lower than in the IRs. Overall, 128 genes were annotated in the 17 *Viola* species, of which 110 were unique, consisting of 76 protein-coding, 30 tRNA, and four rRNA genes (Table 1). The encoded genes of the cp genomes are divided into four categories based on their functions: photosynthesis genes, self-replication genes, other biosynthesis genes, and some genes of unknown function (Table S1). Among these genes, three genes (*infA*, *rpl32*, *rps16*) were completely degraded. There were 17 intron-containing genes in each of the *Viola* species, of which two PCGs (*ycf3* and *clpP*) had two introns, 9 PCGs (*atpF*, *ndhA*, *ndhB*, *petB*, *petD*, *rpl2*, *rpl16*, *rpoC1*, and *rps12*) and 6 tRNAs (*trnA-UGC*, *trnG-UCC*, *trnI-GAU*, *trnK-UUU*, *trnL-UAA*, and *trnV-UAC*) had a single intron each.

**Table 1:** The cp genome characteristics of 17 *Viola* species

Species	Raw reads (pair)	Genome Size (bp)	LSC region (bp)	IR region (bp)	SSC region (bp)	GC content (%)				Number of Genes			
						Over all	LSC	IR	SSC	Total	CDS	tRNAs	rRNAs
*Viola acuminata*	9,709,090	158940	87250	27159	17372	36.1	33.6	42.1	29.8	110	76	30	4
*V. chaerophylloides*	8,709,947	157113	85577	27154	17228	36.3	34.0	42.2	29.9	110	76	30	4
*V. collina*	7,447,895	157890	86495	27102	17191	36.3	33.8	42.2	30.1	110	76	30	4
*V. dissecta*	7,178,430	157113	85557	27154	17228	36.3	34.0	42.2	29.9	110	76	30	4
*V. inconspicua*	6,817,359	156624	85822	27122	16558	36.3	33.8	42.1	30.1	110	76	30	4
*V. mirabilis*	-	158162	86565	27123	17351	36.2	33.8	42.2	29.9	110	76	30	4
*V. monbeigii*	7,091,420	156580	85726	26423	18008	36.3	33.8	42.6	29.6	110	76	30	4
*V. mongolica* 1	9,917,465	156870	85364	27184	17138	36.4	34.0	42.1	30.0	110	76	30	4
*V. mongolica* 2	7,985,972	156904	85373	27192	17147	36.4	34.0	42.1	30.0	110	76	30	4
*V. patrinii*	8,016,998	156508	85694	27404	18006	36.3	33.8	42.6	29.6	110	76	30	4
*V. phalacrocarpa*	1,630,516	156483	85669	27404	18006	36.3	33.8	42.6	29.6	110	76	30	4
*V. philippica* 1	8,268,704	156599	85751	26422	18004	36.2	33.8	42.6	29.6	110	76	30	4
*V. philippica* 2	5,611,822	157298	85756	27195	17146	36.2	33.8	42.1	29.9	110	76	30	4
*V. prionantha*	8,175,231	156509	85693	26404	18008	36.3	33.8	42.6	29.6	110	76	30	4
*V. raddeana*	-	157597	86460	26924	17289	36.2	33.8	42.3	29.9	110	76	30	4
*V. variegata*	7,200,026	156582	85769	26411	17991	36.3	33.8	42.6	29.6	110	76	30	4
*V. websteri*	-	158111	86588	27166	17191	36.2	33.7	42.1	29.9	110	76	30	4
*V. yezoensis*	4,636,893	156537	85706	26417	17997	36.3	33.8	42.6	29.6	110	76	30	4
*V. yunnanfuensis* 1	6,179,277	156974	85975	27160	16679	36.3	33.8	42.1	30.2	110	76	30	4
*V. yunnanfuensis* 2	4,905,794	156931	85858	27208	16663	36.3	33.8	42.1	30.2	110	76	30	4

*LSC* Large single copy, *SSC* Small single copy, *IR* Inverted repeat, *tRNA* transfer RNA, *rRNA* ribosomal RNA

### Structural comparison of *Viola* cp genomes

The locations of IR/SC junctions were conserved among all cp genomes (Fig. S1). In general, the *rps19* gene extended 2-73 bp into the IRs at the junction of the LSC/IRb (JLB), resulting in the duplication of 3’-ends of this gene in the IRa region. The *ndhF* gene was located in the SSC at a 3-33 bp distance from the SSC/IRb (JSB) border. Meanwhile, in *V. mongolica* and *V. yunnanfuensis* the *ndhF* gene traverses the JSB by extending 55 and 51 bp, respectively, into the IRb region. In *V. philippica*, the *ndhF* gene was located in the SSC at a 33 bp distance from the SSC/IRb (JSB) border. In all cp genomes, the SSC/IRa (JSA) junction was located within the *ycf1* gene, with 1091 to 1866 bp of *ycf1* duplicated in the IRb region. In addition, the *trnH* genes were all located in the LSC region, with the distance between *trnH-GUG* and the LSC/IRa (JLA) border varying from 26 to 85 bp.

The number of repeat sequences was calculated and the threshold of repeat length was set to ≥30 bp. A total of 729 repeats were detected in the 17 *Viola* cp genomes (Fig. S2A). The results revealed that *V. acuminata*, *V. mirabilis*, and *V. raddeana* had the greatest number of repeats (49), while *V. mongolica* had the least (33). Conversely, such complementary repeats were not detected in the cp genomes of *V. mongolica* and *V. yezoensis*. Additionally, tandem repeats that ranged from 30 to 33 bp were the most abundant, followed by those that ranged from 38 to 41 bp. The number of repeats in *V. philippica* was 46, comprising 19 forward repeats, 8 reverse repeats, 1 complementary repeat, and 18 palindromic repeats (Fig. S2B). A total of 450 SSRs were identified; the most abundant repeats in *Viola* cp genomes were mononucleotide repeats, followed by di-, and tetra- nucleotide repeats (Table S2). The most common mononucleotide repeat type was A/T, and all the dinucleotide repeats were composed of AT/TA. Meanwhile, tetranucleotide repeats were detected only in the cp genome of *V. websteri*. These SSRs, were located most often in the LSC regions. We identified 22 SSRs in the cp genome of *V. philippica*, including 16 mononucleotide repeats (15 A/T repeats and 1 G/C repeats) and 6 dinucleotide repeats (2 AT repeats and 4 TA repeats).

### Identification of hypervariable regions

**Fig. 2:**
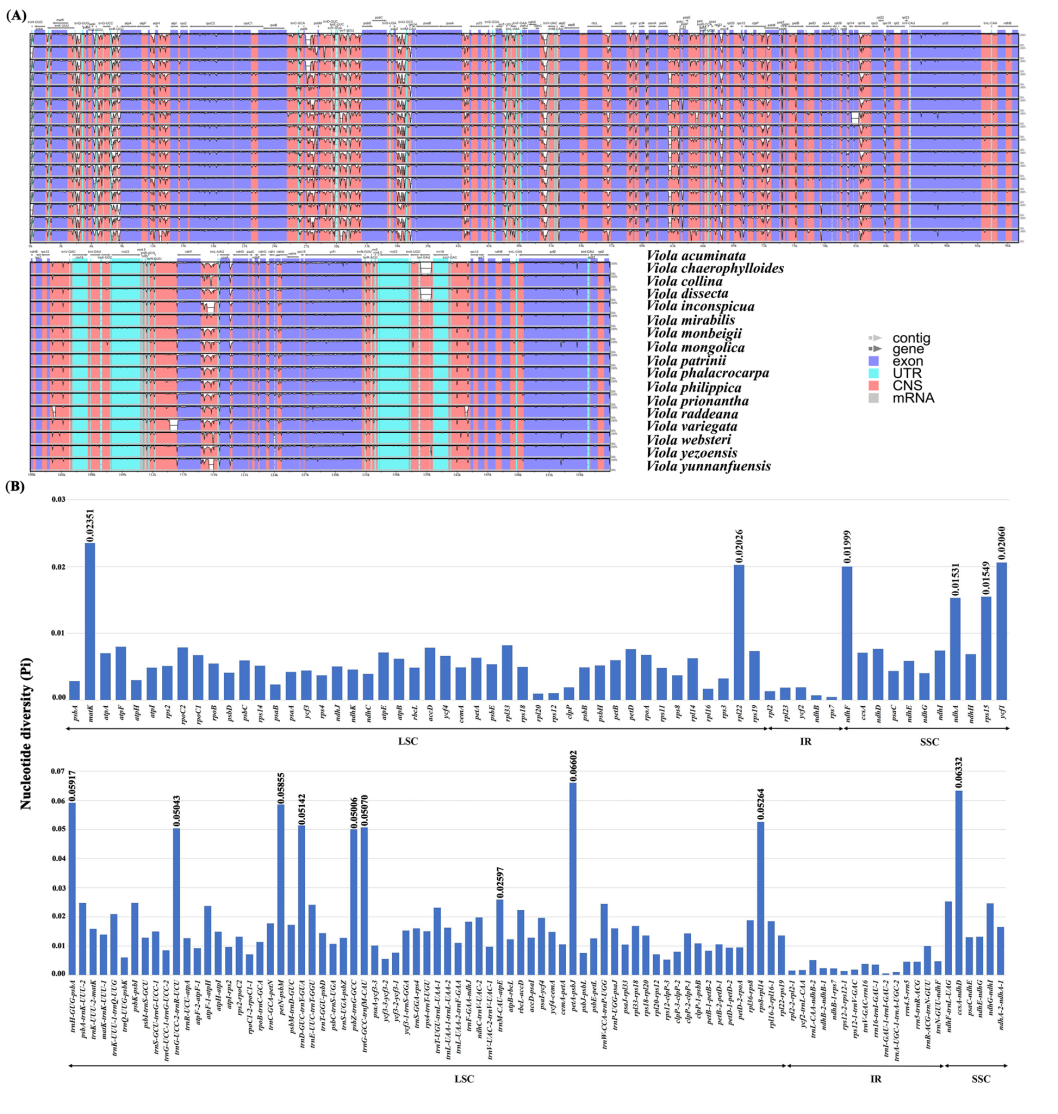
Sequence polymorphism among 17 *Viola* cp genomes. **A** Sequence alignment of *Viola* cp genomes, with *Viola acuminata* as the reference using mVISTA. The position and transcriptional direction of each gene are indicated by gray arrows. The vertical scale indicates the percentage of identity, ranging from 50 to 100%. The horizontal axis indicates the coordinates within the chloroplast genome. Genome regions are color-coded as exons, introns, and intergenic spacer (IGS). **B** The nucleotide diversity (Pi) values of the 17 *Viola* cp genomes; Pi values of coding genes and Pi values of IGS

High levels of sequence similarity was identified across all 17 *Viola* species. However, it was observed that the LSC and SSC regions were more divergent than IRs. In addition, intergenic regions exhibit greater divergence than coding regions (Fig. 2A). The nucleotide diversity (Pi) value was calculated, and their values varied from 0 to 0.06602 (Fig. 2B). High sequence divergence was detected in the following genomic regions: *matK*, *ndhF*, *ycf1*, *rpl22*, *rps15*, *ndhA*, *petN-psbM*, *petA-psbJ*, *ccsA-ndhD*, *trnG-UCC-2-trnR-UCU*, *rps8-rpl14*, *trnD-GUC-trnY-GUA*, *trnG-GCC-trnfM-CAU*, *trnH-GUG-psbA*, *psbZ-trnG-GCC*, and *rbcL-accD*. These divergent regions could be candidates for the development of critical molecular markers for phylogenetic analyses of *Viola* species.

### Chloroplast genome for identification of *V. philippica*

The complete cp genome and 16 highly divergent regions (*matK*, *ndhF*, *ycf1*, *rpl22*, *rps15*, *ndhA*, *petN-psbM*, *petA-psbJ*, *ccsA-ndhD*, *trnG-UCC-2-trnR-UCU*, *rps8-rpl14*, *trnD-GUC-trnY-GUA*, *trnG-GCC-trnfM-CAU*, *trnH-GUG-psbA*, *psbZ-trnG-GCC*, and *rbcL-accD*) generated phylogenetic trees with strong support (Fig. S3). Zi Hua Di Ding 1 and Zi Hua Di Ding 2 clustered with all *Viola* species, not *Corydalis tomentella* or *Oxytropis arctobia*. The results showed that “Zi Hua Di Ding 1” formed a clade with *V. yezoensis* and not *V. philippica*. Zi Hua Di Ding 2 is nested between *V. philippica* 1 and *V. philippica* 2.

**Fig. 3:**
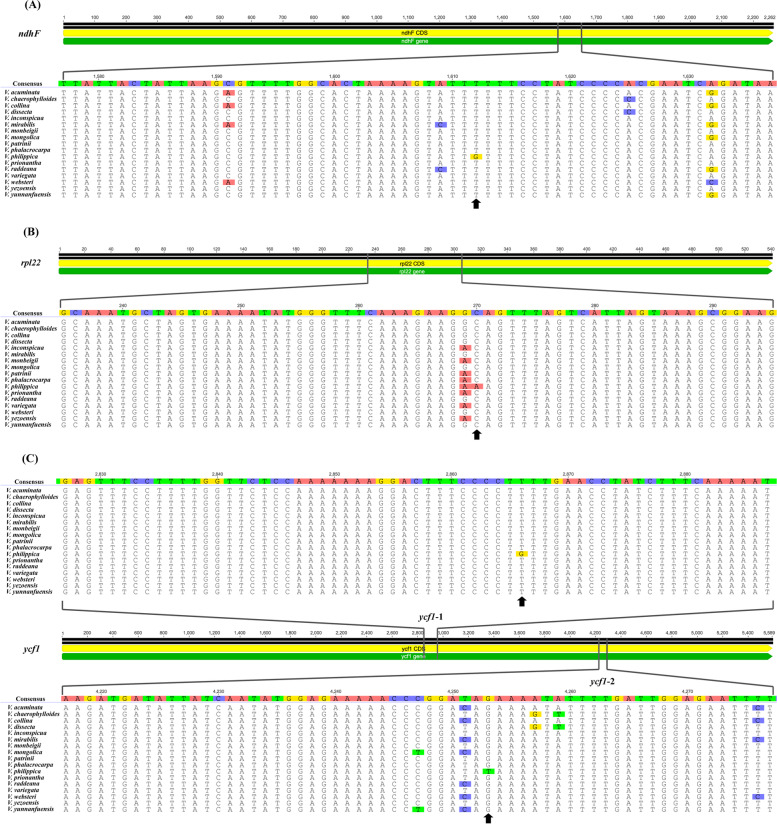
Unique variation sites in *ndhF*, *rpl22*, and *ycf1* of *Viola philippica*. Alignment of four fragment sequences of 17 *Viola* species. **A***ndhF*, (**B**) *rpl22*, (**C**) *ycf1*. The black arrow at the bottom indicated four species-specific variation sites in *Viola philippica*

**Fig. 4:**
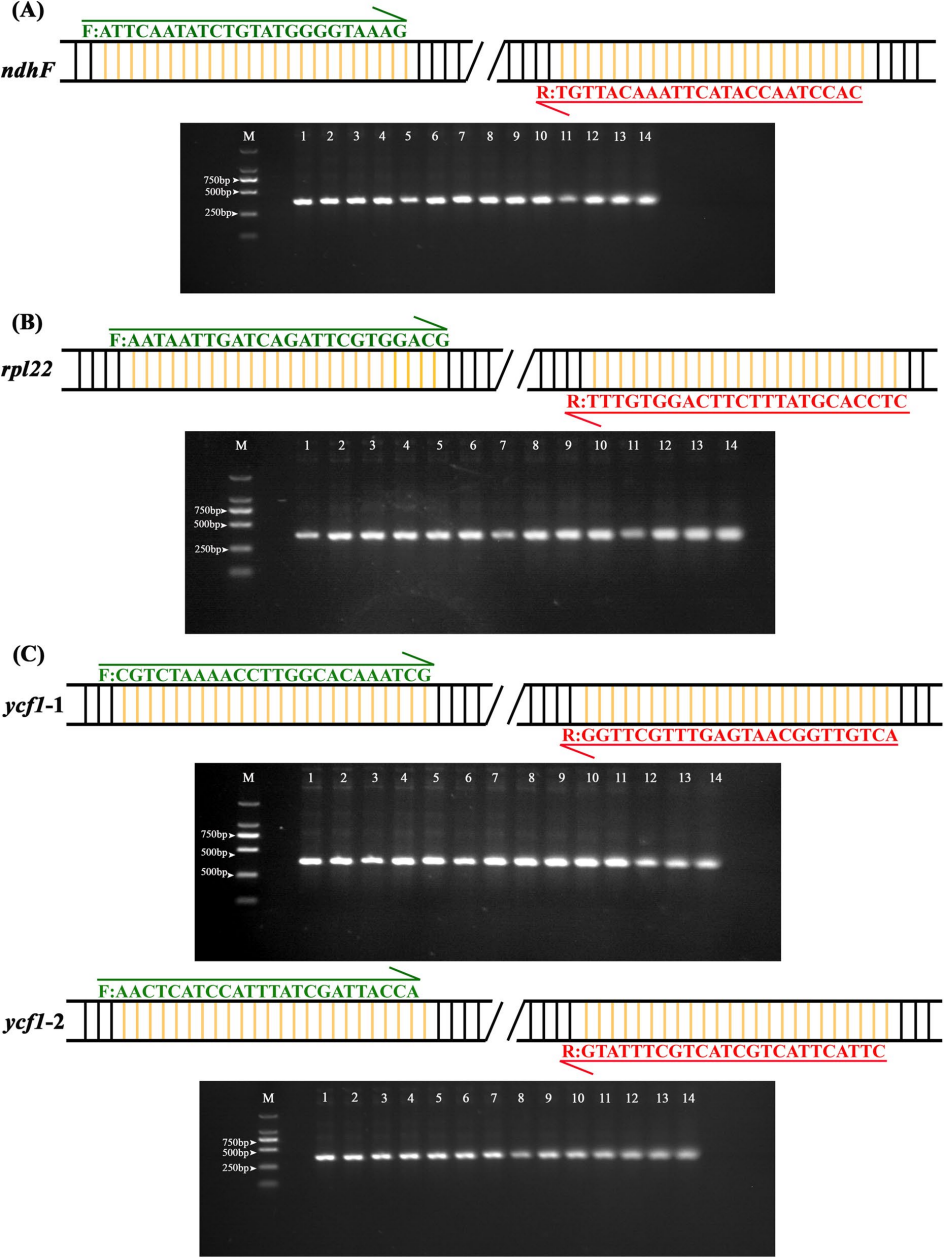
The primer sequences and electrophoretogram for four sequence fragments with unique variation sites in 14 newly sequenced *Viola* species. **A***ndhF*, (**B**) *rpl22*, (**C**) *ycf1* primer pairs. Left and right arrows indicate forward and reverse primers, respectively. M: Marker. 1–14: *V*. *acuminata*; *V*. *chaerophylloides*; *V*. *collina*; *V*. *dissecta*; *V****.****inconspicua*; *V*. *monbeigii*; *V*. *mongolica*; *V*. *patrinii*; *V. phalacrocarpa*; *V*. *philippica*; *V*. *prionantha*; *V*. *variegate*; *V*. *yezoensis*; *V*. *yunnanfuensis*

In this study, four species-specific variable sites were present in *ndhF*, *rpl22*, and *ycf1* of *V. philippica* in comparison to other *Viola* species. The first unique locus is at positions 1612 of *ndhF* in *V. philippica*, where a G is located instead of a T. The second unique locus is A at position 270 of *rpl22* in *V. philippica*, while in other species there is a C. The other two unique sites are G and T at positions 2839 and 4217 of *ycf1* in *V. philippica*, in other species these sites are populated by T and G, respectively (Fig. 3)*.* The nucleotide sequences of four pairs of specific primers used for PCR validation in 14 species are shown in Fig. 4. The amplified products for all individuals were approximately 350 bp (Fig. 4). Nucleotides sequences were consistent between Sanger sequencing results and next-generation sequencing results.

### Infrageneric relationships of *Viola*

**Fig. 5:**
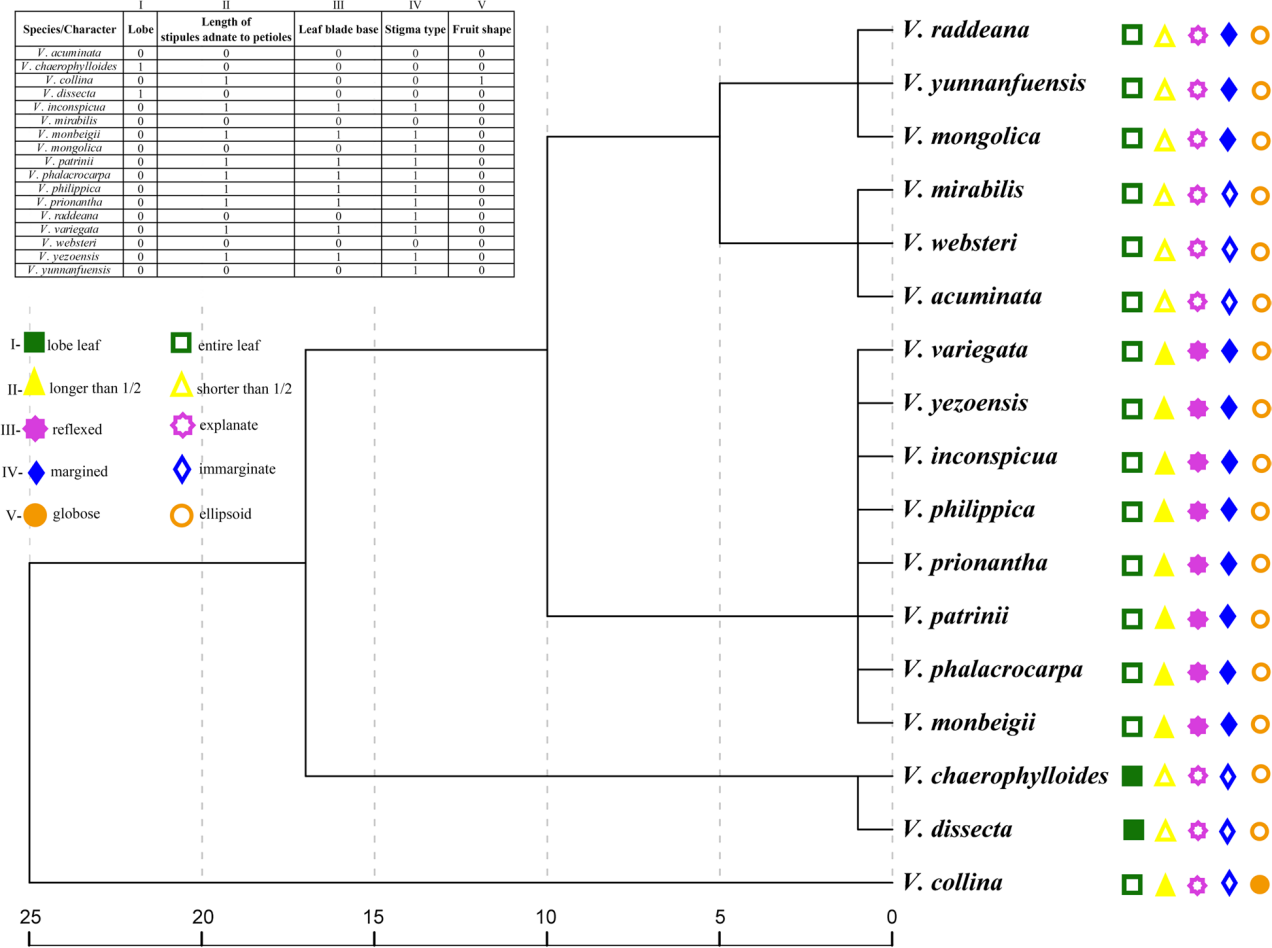
Morphological clustering of 17 *Viola* species. Top left inset shows cods of five morphological characters in 17 *Viola* species. Lobe: 0 = entire leaf, 1 = lobed leaf; Length of stipules adnate to petioles: 0 = stipule adnate with petioles shorter than one-half, 1 = stipule adnate with petioles longer than one-half; Leaf blade baes: 0 = explanate, 1 = reflexed; Stigma type: 0 = immarginate, 1 = margined; Fruit shape: 0 = capsule ellipsoid, 1 = capsule globose. Each trait was represented by a different shape on the cluster analysis tree. Use solid or hollow to indicate different types

**Fig. 6:**
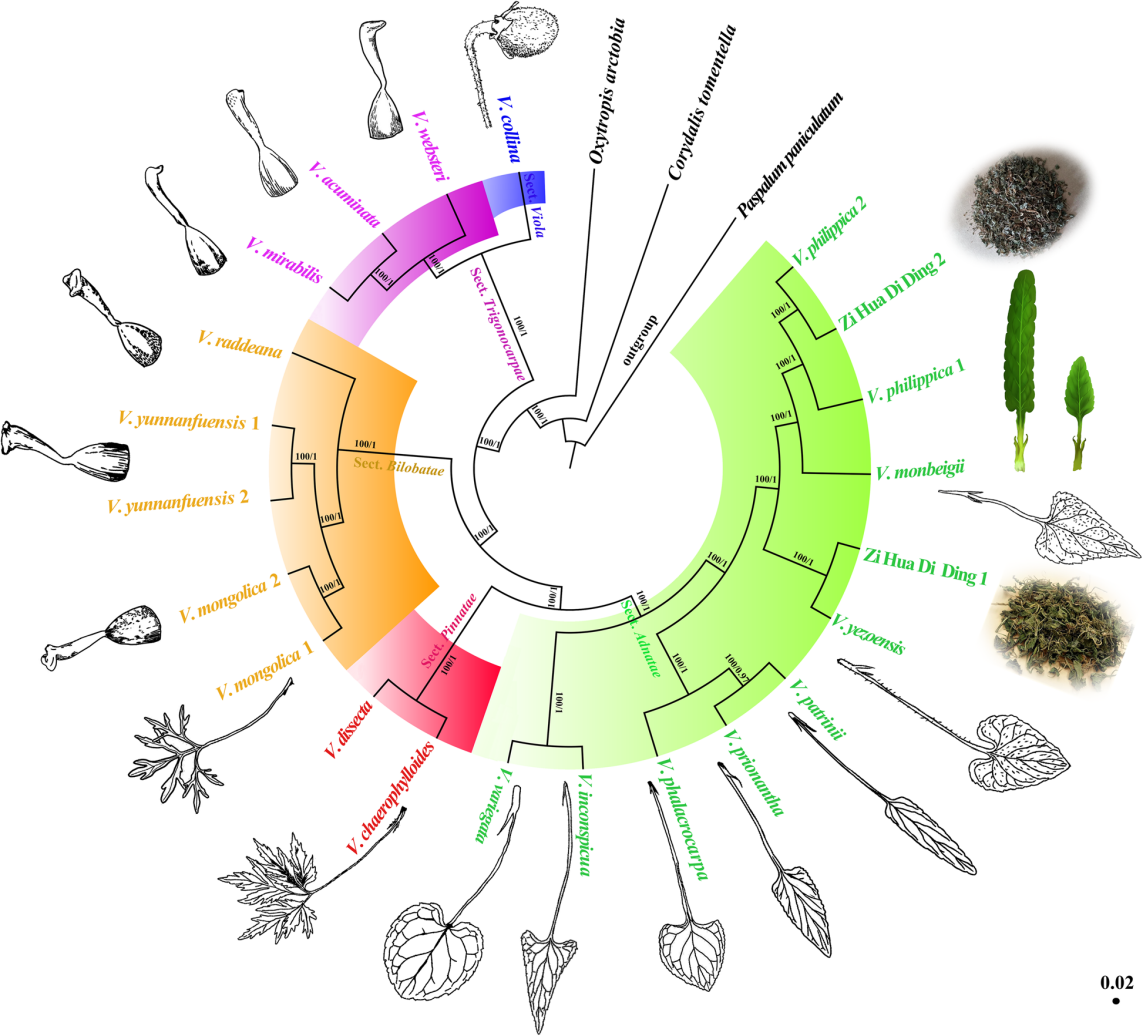
Maximum Likelihood (ML) and Bayesian Inference (BI) phylogenetic trees based on whole cp genomes of 24 samples. *Paspalum paniculatum* was set as the outgroup. The numbers above the branches represent ML bootstrap values/BI posterior probabilities. The shared morphological characteristics for each of the five clades were shown next to each species in the way of hand-drawn illustrations, and the dry leaves of “ZHDD” bought from TCM pharmacy were shown next to “Zi Hua Di Ding1, 2”. The hand-drawn illustrations credits: Dong-Ling Cao. In the mature period with dimorphic leaf blades, i.e., smaller triangular-ovate for the lower leaf blades and longer oblong-ovate for the upper leaf blades was shown next to *V. philippica*. For section *Viola*, the fruit is the globose capsule. For section *Trigonocarpae*, the stigmas are clavate, slightly curved forward at the base, and the apex is uncinate or papillose; For section *Bilobatae*, the stigmas are 2-lobed, thick, with a conspicuous stigma hole; For section *Pinnatae*, the leaf blades are lobe; For section *Adnatae*, the length of stipules adnate with petioles is longer than one-half length of stipules

Comparison of morphological characteristics such as lobe vs entire, length of stipule adnate with petioles, the shape of the leaf blade, stigma type, and fruit shape, demonstrated that the 17 species could be classified into five clades including sections *Viola*, *Pinnatae*, *Adnatae*, *Trigonocarpae*, and *Bilobatae* (Fig. 5). Based on the topologies of all ML and BI trees, all 17 species were further divided into these five monophyletic groups (Fig. 6). Notably, when comparing the morphological and the cp genome tree, the species in all five clades were identical. We summarized shared morphological characteristics of species belonging to the same clade by comparative morphological analysis. The shared morphological characteristics for each of the five clades were shown next to each species in hand-drawn illustrations, and the dry leaves of “Zi Hua Di Ding” purchased from the TCM pharmacy were shown next to Zi Hua Di Ding 1, 2 (Fig. 6). For sections *Viola*, *Trigonocarpae*, *Bilobatae*, *Pinnatae*, and *Adnatae*, the specific globose capsule, shared beak stigma, shared 2-lobed stigma, unique dissected leaf blade, and stipule adnate with petioles longer than one-half its length were the main morphological characteristics of each section, respectively. Importantly, the dimorphic leaf blade during the flowering period, i.e., smaller triangular-ovate for the lower leaf blades, longer oblong-ovate for the upper leaf blades, and fine tubular calcar with slightly downward curved ends were the most important morphological character for the identification of *V. philippica* (Fig. 1A, B).

The topologies of the ML and BI trees were highly concordant for complete cp genome sequences and highly diverged sequences with high support (Fig. 6, S5). In the present study, *Viola* was monophyletic and formed five clades. For section *Viola*, *V*. *collina* occupied the most basal position. The clade comprising *V. acuminata*, *V. mirabilis*, and *V. websteri* was located within section *Trigonocarpae.* For section *Bilobatae*, the species *V. mongolica* and *V. yunnanfuensis* were sisters to *V. raddeana*. For section *Pinnatae*, *V*. *chaerophylloides* is sister to *V*. *dissecta.* In section *Adnatae*, *V*. *inconspicua* and *V*. *variegate* firstly diverged, and *V*. *phalacrocarpa*, *V*. *patrinii*, and *V*. *prionantha* were sisters to *V*. *yezoensis*, *V*. *monbeigii*, and *V*. *philippica*.

## Discussion

### Cp genome structural changes in *Viola*

The cp genomes of land plants are highly conserved and can therefore provide important phylogenetic data. The cp genomes of 17 *Viola* species exhibited a typical quadripartite structure, with an identical number of protein-coding genes, tRNAs, and rRNAs (Table 1), consistent with the previously reported *Viola* cp genome [43, 44]. Furthermore, we identified that the genes *infA*, *rpl32*, and *rps16* were not present in 17 *Viola* cp genomes (Table S1). Studies suggest that the cause of the loss of these genes may be due to parallel loss of chloroplast DNA during the evolution of angiosperms [45–47]. There were 17 intron-containing genes in each *Viola* species analyzed (Table S1). Introns have been reported to increase the transcriptional efficiency of numerous genes in a variety of organisms [48, 49]. Although generally IRs are highly conserved, the expansion and contraction of the IRs is a common characteristic of cp genomes and is thought to be the main cause of their variability in size [50, 51]. These changes have been associated with gene duplication at the junction between the IRs and the LSC and SSC regions, which results in gene content variation between species. The IR expansion of *V. mongolica* and *V. yunnanfuensis* caused the *ndhF* gene into the IRb region. Repeat sequences are not only hotspots for mutations such as nucleotide substitutions, insertions and deletions, but are also important in phylogenetic studies [52, 53]. The number of repeats in *V*. *philippica* was 46, with 19 forward repeats, 8 reverse repeats, 1 complementary repeat, and 18 palindromic repeats. These data will provide a basis for studying the phylogeny of *V. philippica*. Notably, most mononucleotides and dinucleotides are composed of A and T, which may contribute to a bias in base composition [54].

### Identification of *V. philippica* by morphology, cp genome phylogeny, and species-specific variable sites

The safe use of TCM conventionally relies on correct identification and TCM-guided clinical prescription. In the trade of herbal medicines, consumers often do not verify the identity original voucher specimen. Therefore, ensuring the authenticity of raw materials used as herbs is particularly important [55]. The traditional identifications of herbs rely upon morphology, odor, or flavor and is performed by experts. Even now, morphological characteristics are still an important basis for identification [56–58]. However, it is difficult to train a person to acquire the required professional skills. Moreover, for some specimens with very similar morphological characteristics, it is difficult even for experts to accurately identify. It is well known that *V*. *philippica* and closely related species have highly similar morphological characters. The identification of common herbs methods including morphological observation, thin layer chromatography (TLC), high-performance liquid chromatography (HPLC), near-infrared spectroscopy (NIRS), and metabolomic approaches [59–62]. However, these methods are often complicated and costly. DNA barcoding can achieve rapid, accurate, and automated species identification [63–65]. ITS and cp genome fragment analysis have been used to distinguish *V. philippica* from closely related species previously, however, fewer adulterants of *V. philippica* were sampled and strong support for some nodes in the phylogenetic tree was not acquired [28, 30]. *Viola* exhibit a low level of genetic differentiation as revealed by the ITS analysis [66]. However, ITS and plastid datasets did not provide substantial phylogenetic information, and the phylogenetic position of species within the genus is uncertain. Due to the rapid development of sequencing techniques and bioinformatics, the complete cp genomes of plants can be rapidly acquired at low cost. Cp genomes have a moderate rate of nucleotide evolution, which results in their suitability for species identification and phylogenetic studies at different taxonomic levels [67, 68]. Cp genomes are proposed as potential super-barcode for species identification [69–71]. The *V. philippica* were analyzed by Blast online comparison in the NCBI database. The results of gene sequence similarity comparison showed that the *V. philippica* sequences in this study had highest homology with *V. philippica* sequences that had been registered on GenBank. The similarity results reach more than 98%. This suggests that validation of the chloroplast genome is effective. The use of genetic distances using standardized gene regions (DNA barcodes) has provided complementary or alternative support for species identification, which is especially useful when distinct morphological characters are scarce or subtle [72]. Here, the genetic distance results based on the complete chloroplast genome showed that genetic distance is small with 17 *Viola* species. Among them, *V. philippica* and *V. monbeigii* had the smallest genetic distance, indicating that they are both closely related, which is consistent with the phylogenetic tree results. The identity of genes between populations within species is quite high, and consequently, genetic distance is small (Table S3). In our study, all common adulterants of *V. philippica* were sampled. The topologies of ML and BI trees were highly concordant for the complete cp genome sequence and the highly diverged sequences. Our phylogenetic results indicated strong support for *Viola* species in all sample, and the cp genomes sequences could be used as a super-barcode for authentication of *V. philippica* (Fig. 6). There are four specific variable sites in *ndhF*, *rpl22*, and *ycf1* of *V. philippica*, which were identified when we compared the alignment matrix of these genes for all sampled *Viola* species. Furthermore, Sanger sequencing results validated the four unique variable sites of *V. philippica* (Fig. 3), suggesting that they can be selectively amplified to distinguish *V. philippica* from other sampled *Viola* species, especially its adulterants. In short, the original source plant of “Zi Hua Di Ding” can be accurately identified by these species-specific variable sites. *Viola* species are traditionally morphologically difficult taxon to classify due to their very similar morphological characteristics. The wide distribution coupled with frequent hybridization increases the difficulty of species identification and phylogenetic analysis. Geographical differences and frequent hybridization may cause the occurrence of interspecific mutations in some genes of *V. philippica* (Fig. S4). In practice, these four unique variable loci should be considered simultaneously to ensure the greatest possible accuracy of identification. In addition, important morphological characteristics and the construction of a phylogenetic tree are presented to aid in the accurate identification of *V. philippica*. We applied these methods to examine two “Zi Hua Di Ding” purchased randomly from a local TCM pharmacy. The results showed the “Zi Hua Di Ding 1” and *V. yezoensis* clustered together, but not with *V. philippica*. “Zi Hua Di Ding 2” is nested between *V. philippica* 1 and *V. philippica* 2. There is a high probability that the “Zi Hua Di Ding 1” purchased from TCM pharmacy in this study was not *V. philippica*, and considered should be *V. philippica* adulterants. “Zi Hua Di Ding 2” should be considered as genuine *V. philippica*.

In addition, morphological comparative analysis of the dimorphic leaf blade during the flowering period, i.e., smaller triangular-ovate for the lower leaf blades and longer oblong-ovate for the upper leaf blades in addition to fine tubular calcar with slightly downward curved ends were the most important morphological characteristic for the identification of *V*. *philippica*. In addition, we observed slight morphological changes in *V. philippica* individuals sampled during different growth periods. For example, some flowers become pale in color and the base of leaves occasionally widen. Therefore, we propose the flowering period to be the optimal time to morphologically identify *V*. *philippica*. According to reports regarding *V. philippica* extracts, the medicinal components vary at different times of the year, and the appropriate time of collection can be chosen according to medicinal needs [73]. Overall, morphological characteristics, cp genome phylogeny and species-specific variable sites analysis can be applied to distinguish *V. philippica* from other sample *Viola* species. For the authentication of “Zi Hua Di Ding”, a combination of method can be chosen, so that *V. philippica* can be accurately distinguished from its common adulterants. The identification of *V. philippica* is carried out according to the actual situation, combined with the distinct methods.

### Infrageneric relationships of *Viola* (section *Adnetae*, *Pinnatae*, *Bilobatae*) based on morphological characters and cp genomes

Chloroplast genome sequences are invaluable for understanding plant evolution and phylogeny [74, 75]. One or a more of several chloroplast molecular markers (*atpB-rbcL*, *matk*, *petG*-*trnW*, *psbA-trnH*, *psbZ-trnG*, *psbK-I*, *rps19-trnH*, *rpl16-rps3*, *rpl2-23*, and *trnL-F*) and nuclear ITS were used to infer the phylogeny of *Viola*, however, the majority of interspecies relationships are not currently well resolved [27–29, 76]. The short branches in the phylogenetic tree in our study show consistency with previous studies and are the result of rapid divergence (Fig. S5) [66]. Their sequence identity is therefore likely to reflect explosive radiation, and not simply a recent origin. *Viola* species are traditionally morphologically difficult taxon to classify due to their very similar morphological characteristics, and there is some synapomorphy in *Viola* species. This may be why phylogenetic analyses of *Viola* based on molecular inference are often inconsistent with the results of the traditional morphological study. Complete cp genomes contain a wealth of genetic variation and is an ideal source of data to study phylogeny among species [77–79]. Based on the whole cp genome, the infrageneric phylogenetic relationships of *Viola* were resolved in this study, and both ML and BI trees were strongly supported. Therefore, we suggested that the complete cp genome can be used as a super-barcode to distinguish closely related *Viola* species. Furthermore, relationships among sampled *Viola* species were also resolved based on only 16 molecular markers with high Pi values, their topologies are highly consistent with the complete cp genome. Therefore, these identified regions (*matK*, *ndhF*, *ycf1*, *rpl22*, *rps15*, *ndhA*, *petN-psbM*, *petA-psbJ*, *ccsA-ndhD*, *trnG-UCC-2-trnR-UCU*, *rps8-rpl14*, *trnD-GUC-trnY-GUA*, *trnG-GCC-trnfM-CAU*, *trnH-GUG-psbA*, *psbZ-trnG-GCC*, and *rbcL-accD*) could be used as markers for elucidating phylogenetic relationship within *Viola* species*.* These findings provide additional information for the selection of effective molecular markers to detect intra- and interspecific genetic polymorphisms (Fig. S3).

*Viola* are known as one of the taxonomically difficult groups to define since *Viola* species possessing many morphologically similar characteristics and intermediate forms that occur freely due to interspecific hybridization [23–25]. Morphological data are necessary to identify species and to infer their relationships in phylogenetic studies [80–82]. Interestingly, in this study the position of species in each of the five clades were identical between the morphological tree and the cp genome tree. The results revealed that species with the same morphological characteristics cluster together with a higher internal resolution (Fig. 5). Therefore, we advocated that the cp genomes should be combined with morphological characteristics in analyze of the phylogenetic position and identification of *V. philippica*. Previous research reported that *V. chaerophylloides* and *V. dissecta* are distantly related and that section *Pinnatae* should be treated as a subsection under section *Adnatae* [29, 30]. However, the leaf blade of *V. chaerophylloides* and *V. dissecta* were pinnatifid and parted, respectively, and it has been demonstrated that the lobe is not a taxonomic characteristic in the *Viola.* In this study, we identified a close relationship between *V. chaerophylloides* and *V. dissecta*; they form a clade with strong node support. This data indicates that the lobe can be used as a classification characteristic for *Viola*. These data support the taxonomic position of section *Pinnatae*. Previous study suggested that *V. mongolica* and *V. yunnanfuensis* belong to the section *Adnatae* according to the morphological characters of the stipules [29]. However, in our study, the clade composed of *V. mongolica* and *V. yunnanfuensis* was a sister to that *V. raddeana*, and they share the bilobate stigma. Therefore, we suggest that *V. mongolica* and *V. yunnanfuensis* are positioned within section *Bilobatae*. In addition, we must note that some species in section *Adnatae*, whose stipules are adnate to the petiole should be carefully examined for their stigma in future studies. Phylogenetic analysis based on the cp genomes successfully resolved the relationship between *Viola* sampled species*.* Due to the overlapped taxonomic characters of sections *Adnatae*, *Bilobatae*, and *Pinnatae*, the ranges need to be further delimitated. It is worth noting that a comprehensive consideration of morphological characters is necessary for the phylogenetic study of *Viola*. The monophyly of sections *Adnatae* and related taxa and its taxonomic position need to be further analyzed, to generate more data we plan to conduct further investigations with broad sampling and further more morphological evidence.

## Conclusion

In the current study, all *Viola* cp genomes share a highly similar gene content and order. The topologies of ML and BI trees are highly concordant for both complete cp genome sequences and highly diverged sequences. Phylogenetic analysis revealed highly supported for interspecies relationships. Morphological characteristics, cp genome phylogeny, and species-specific variable sites can be applied to distinguish *V*. *philippica*, the only source plant of “Zi Hua Di Ding”, from other *Viola* species, in particular its adulterants. Furthermore, we propose that the most favorable time for accurate identification of *V*. *philippica* is the flowering period. This study provides invaluable data for the improvement of species identification and germplasm of *V. philippica* that may facilitate the application of a super-barcode in TCM identification and enable future studies on phylogenetic evolution and safe medical applications.

## Methods

### Sample collection, DNA extraction, and sequencing

The plastomes of 19 samples were newly sequenced in this study, plus 5 plastid genomes already available from GenBank (https://www.ncbi.nlm.nih.gov), for a total of 24 individuals. Plant materials used in this study were collected and deposited at the herbarium of the College of Life Sciences, Shandong Normal University. The sampling newly sequenced species were collected from Shandong Province, China. FSJ and ZXJ undertook the formal identification of the samples (Table 2). No specific permissions were required for the relevant locations/activities and met local policy requirements. Table 2 indicates the detailed voucher and locality information for the newly sequenced species. Total genomic DNA was extracted using a modified cetyltrimethylammonium bromide (CTAB) method [83]. The quality and concentration of the genomic DNA were checked using 1.5% agarose gel electrophoresis and the NanoDrop 2000c spectrophotometer (Thermo Fisher Scientific Inc., USA). The total genomic DNA was used for library preparation and paired-end (PE) sequencing by the Illumina Novaseq instrument at Novogene (Beijing, China). The raw data is approximately 2Gb and the insert library size is approximately 350bp. In addition, two individuals for each species of *V. philippica*, *V. mongolica* and *V. yunnanfuensis* were sampled from different locations and labeled *V. philippica* 1, *V. philippica* 2, *V. mongolica* 1, *V. mongolica* 2, *V. yunnanfuensis* 1, and *V. yunnanfuensis* 2 (Fig. 6).

**Table 2:** Summary of the sequencing data for 17 *Viola* species

Species	Collecting locations	GPS	Voucher specimen number	GenBank accession number
*Viola acuminata* Ledeb.	Zibo, Shandong, China	117°50'E, 36°29'N	SDNU 042910	MW802528
*V. chaerophylloides* (Regel) W. Becker	Zibo, Shandong, China	117°52'E, 36°19'N	SDNU 042915	MW802529
*V. collina* Besser	Zibo, Shandong, China	117°51'E, 36°29'N	SDNU 042917	MW802530
*V. dissecta* Ledeb.	Zibo, Shandong, China	117°53'E, 36°25'N	SDNU 042922	MW802531
*V. inconspicua* Blume	Zibo, Shandong, China	117°51'E, 36°20'N	SDNU 091832	MW802532
*V. mirabilis* L.	-	-	-	NC041582
*V. monbeigii* W.Becker	Qingdao, Shandong, China	120°28'E, 36°06'N	SDNU 3157	MW802533
*V. mongolica* Franch. 1	Zibo, Shandong, China	117°51'E, 36°29'N	SDNU 050113	MW802534
*V. mongolica* Franch. 2	Jinan, Shandong, China	117°15'E, 36°55'N	SDNU 706	ON548135
*V. patrinii* Ging.	Zibo, Shandong, China	117°51'E, 36°29'N	SDNU 042913	MW802535
*V. phalacrocarpa* Maxim.	Yantai, Shandong, China	121°16'E, 37°29'N	SDNU 890210	MW802536
*V. philippica* Cav. 1	Jinan, Shandong, China	116°50'E, 36°32'N	SDNU 89588	MZ343563
*V. philippica* Cav. 2	Jinan, Shandong, China	117°04'E,36°65'N	SDNU 704	ON548136
*V. prionantha* Bunge	Zibo, Shandong, China	117°51'E, 36°29'N	SDNU 091825	MW802538
*V. raddeana* Regel	-	-	-	NC041584
*V. variegata* Fisch. ex Link	Zibo, Shandong, China	117°51'E, 36°29'N	SDNU 042916	MW802539
*V. websteri* Hemsl.	-	-	-	NC041585
*V. yezoensis* Maxim.	Qingdao, Shandong, China	120°28'E, 36°06'N	SDNU 3076	MW802540
*V. yunnanfuensis* W. Becker 1	Qingzhou, Shandong, China	118°28'E, 36°41'N	SDNU 0022	MW802541
*V. yunnanfuensis* W. Becker 2	Jinan, Shandong, China	117°04'E, 36°62'N	SDNU 705	ON548137

### Cp genomes assembly and annotation

We assembled the cp genomes by Organelle Genome Assembler (OGA; https://github.com/quxiaojian/OGA) [84]. Annotation was performed by using Plastid Genome Annotator (PGA; https://github.com/quxiaojian/PGA) [85]. Geneious v8.0.2 was used for annotation correction [86]. The circular maps for newly sequenced cp genomes were generated using the OGDRAW v1.3.1 [87]. All chloroplast genomes assembled in this study have been deposited in GenBank under accession numbers of MW802528 - MW802536, MW802538 - MW802541, MZ343563, and ON548135 - ON548137. Complete plastome of three *Viola* species were downloaded from GenBank, including *V. mirabilis* L. (NC_041582), *V. raddeana* Regel (NC_041584), and *V. websteri* Hemsl. (NC_041585) (Table 2). The genome size, GC content, gene number, and intron number of the 17 complete plastome were summarized by using Geneious v8.0.2.

### Expansion and contraction of IRs

The expansion and contraction of IRs were analyzed by IRscope (https://irscope.shinyapps.io/irapp/) [88], coupled with manual modification. In this study, IR borders and neighboring genes were compared for 17 *Viola* species.

### Characteristics of repeat sequences and SSRs

SSR markers are valuable in study of genetic diversity and molecular marker selection. The size and position of the repeat sequences were detected using REPuter (https://bibiserv.cebitec.uni-bielefeld.de/reputer/) [89], including forward, reverse, complement, and palindromic repeats within the cp genomes. The following settings were used: (1) Hamming distance of 3; (2) 90% or greater sequence identity; (3) a minimum repeat size of 30bp. Simple sequence repeats (SSRs) in cp genomes were detected using MISA [90], with repeat units set to ≥10 for mononucleotide, ≥6 for dinucleotide, and ≥5 for trinucleotide, tetranucleotide, pentanucleotide, and hexanucleotide.

### Comparative analysis and divergence hotspot identification

mVISTA (http://genome.lbl.gov/vista/index.shtml) [91] is a commonly used comparative cp genome map-drawing web application, but the input file of mVISTA needs to meet the format requirements. A custom Perl script (https://github.com/quxiaojian/Bioinformatic_Scripts/get_mVISTA_format_from_GenBank_annotation.pl) was used to convert GenBank annotation files to mVISTA format files. Then we aligned the complete cp genomes using the Shuffle-LAGAN mode of mVISTA, with *V. acuminata* as reference.

Single nucleotide polymorphism (SNP) mainly refers to DNA sequence polymorphism caused by single nucleotide variation at the genome level. The percentage of parsimony information sites (Pi) of the coding and intergenic regions were calculated by using DnaSP v6.0 [92]. The screening conditions were as follows: (1) sequence length > 200 bp; (2) variable sites and parsimony information sites > 0.

### Identification of *V. philippica* and phylogenetic analysis

We blast *V. philippica* with the NCBI database (https://blast.ncbi.nlm.nih.gov/Blast.cgi?PROGRAM=blastn&PAGE_TYPE=BlastSearch&LINK_LOC=blasthome) by Blast online. The *Viola* genetic distances were calculated in MEGA, using Kimura’s 2-parameter model [93]. The coding and intergenic sequences were individually aligned using MAFFT v7.313 [94] with default parameters, and then manually edited using Geneious v8.0.2. Some species have been misidentified mainly because their violet-colored flowers are the same color as those of *V. philippica*, e.g., *Corydalis bungeana* or *Gueldenstaedtia verna.* To improve the reliability and accuracy of the identification results, we added closely related species of both, *Corydalis tomentella* (NC*_*060366*)* and *Oxytropis arctobia* (NC_050861). *Paspalum paniculatum* (MF563367) was set as the outgroup. The ML trees were reconstructed by RAxML v8.0.26 with the GTRGAMMA substitution model and 1000 bootstrap replicates [95]. Bayesian inference of phylogeny was explored using the MrBayes v3.1.2 [96]. Phylogenetic trees were reconstructed based on the following data sets: 1) complete cp genome sequences; 2) highly diverged sequences (*matK*, *ndhF*, *ycf1*, *rpl22*, *rps15*, *ndhA*, *petN-psbM*, *petA-psbJ*, *ccsA-ndhD*, *trnG-UCC-2-trnR-UCU*, *rps8-rpl14*, *trnD-GUC-trnY-GUA*, *trnG-GCC-trnfM-CAU*, *trnH-GUG-psbA*, *psbZ-trnG-GCC*, and *rbcL-accD*).

### Selection of species-specific variable sites in protein-coding genes of *V. philippica*

The specific variable sites of protein-coding genes that can distinguish *V. philippica* from other *Viola* species were screened by examining the alignment matrix of 76 genes for 17 *Viola* species. To ensure the accuracy of the four selected specific variation sites in *V. philippica*, Sanger sequencing of PCR amplicons was performed on 14 newly sequenced *Viola* species. Primers were designed using Primer3 v0.4.0 [97]. The coding regions of the *V. philippica* cp genome were used as the templates for primer design. The forward primers for these four fragments were ATTCAATATCTGTATGGGGTAAAG, AATAATTGATCAGATTCGTGGACG, CGTCTAAAACCTTGGCACAAATCG and AACTCATCCATTTATCGATTACCA and the reverse primers were TGTTACAAATTCATACCAATCCAC, TTTGTGGACTTCTTTATGCACCTC, GGTTCGTTTGAGTAACGGTTGTCA and GTATTTCGTCATCGTCATTCATTC, respectively (Fig. 4, S6). The target fragments that span these four specific sites were about 300 bp, respectively. The PCR amplification was conducted in a total volume of 50 μL reaction system containing 3 uL genomic DNA, 2 uL forward primer, 2 uL reverse primer, 4 uL dNTPs (0.4 mM), and 1uL 1.5 units mix including the high-fidelity polymerase ExTaq (TaKaRa). The amplification was carried out with an initial denaturation step at 95 °C for 3 min followed by 35 cycles of denaturation for 30 s at 95 °C, primer annealing for the 40s at 55 °C, and then product extension for 3 min at 72 °C. A final extension step was at 72 °C for 5 min. The PCR products were verified by gel electrophoresis on a 1.5% agarose gel. All sequences were deposited to GenBank (Table S4).

### Morphological anatomy and clustering

We summarized previous studies on the detailed morphological classification of the *Viola* and conducted extensive trait comparisons and statistical analyses, which are presented by hand-drawn ink line diagrams. We mainly focus on traits that were more controversial in previous studies. For example, the lobe or not, length of stipules adnate to petioles, the shape of the leaf blade, stigma type, and fruit shape. The details of plant materials used were observed and measured using microscopes. The statistical analysis was performed using IBM SPSS Statistics v22.0 (SPSS Inc., Chicago, IL, USA). We coded five-character, including lobe, length of stipules adnate to petioles, leaf blade baes, stigma type, and fruit shape. lobe: 0 = entire leaf, 1 = lobed leaf; length of stipules adnate to petioles: 0 = stipule adnate with petioles shorter than 1/2, 1 = stipule adnate with petioles longer than 1/2; leaf blade baes: 0 = explanate, 1 = reflexed; stigma type: 0 = immarginate, 1 = margined; fruit shape: 0 = capsule ellipsoid, 1 = capsule globose. We mapped these traits onto a cluster analysis tree and represented each trait with a different shape (Table S5).

## Supplementary Information

**Additional file 1: Fig. S1.** Comparisons of LSC, SSC, and IR region borders among 17 chloroplast genomes. **Fig. S2.** Repeat sequences analysis of 17 cp genomes. **Fig. S3.** Maximum Likelihood (ML) and Bayesian Inference (BI) phylogenetic trees are based on 16 highly diverged regions. **Fig. S4.** The variable sites in *ndhF*, *rpl22*, and *ycf1* of *Viola philippica*. **Fig. S5.** Maximum Likelihood (ML) and Bayesian Inference (BI) phylogenetic trees are based on complete chloroplast genome. **Fig. S6.** Original electrophoretogram for four sequence fragments with unique variable sites in 14 newly sequenced *Viola* species.

**Additional file 2: Table S1.** A list of genes found in the cp genomes of 17 *Viola* species. **Table S2.** SSR distributed situation in the 17 *Viola* cp genomes. **Table S3.** Results of genetic distance analysis. **Table S4.** Summary of amplified nucleotide sequences and GenBank accession numbers. **Table S5.** Morphological characteristics for analysis.

## Data Availability

The data sets supporting the results of this article are included within the manuscript and its additional files. The complete plastome and amplified nucleotide sequences of 14 *Viola* species was submitted to GenBank (https://www.ncbi.nlm.nih.gov/) (accession numbers: MW802528 - MW802536, MW802538 - MW802541, MZ343563, ON548135 - ON548137, and MZ407852 - MZ407907; see Table 2, S4). All read data are available at the SRA (http://www.ncbi.nlm.nih.gov/bioproject/751372) with the BioProject following accession numbers: PRJNA751372. These data will remain private until the related manuscript has been accepted. All other data generated in this manuscript are available from the corresponding author upon reasonable request.

## Abbreviations

TCM: Traditional Chinese Medicine
RSV: Respiratory syncytial virus
ITS: Nuclear intergenic transcribed spacer
ISSR: Inter-simple sequence repeat
LSC: Large single-copy
SSC: Small single copy
IR: Inverted repeat
CTAB: Cetyltrimethylammonium bromide
SNP: Single nucleotide polymorphism
SSRs: Simple sequence repeats
Pi: Nucleotide diversity
