# Supplementary figures and images for: Application of chloroplast genome in the identification of Traditional Chinese Medicine Viola philippica
Source: BMC Genomics. 2022 Jul 27;23:540. doi: 10.1186/s12864-022-08727-x (PMC9327190; doi:10.1186/s12864-022-08727-x)

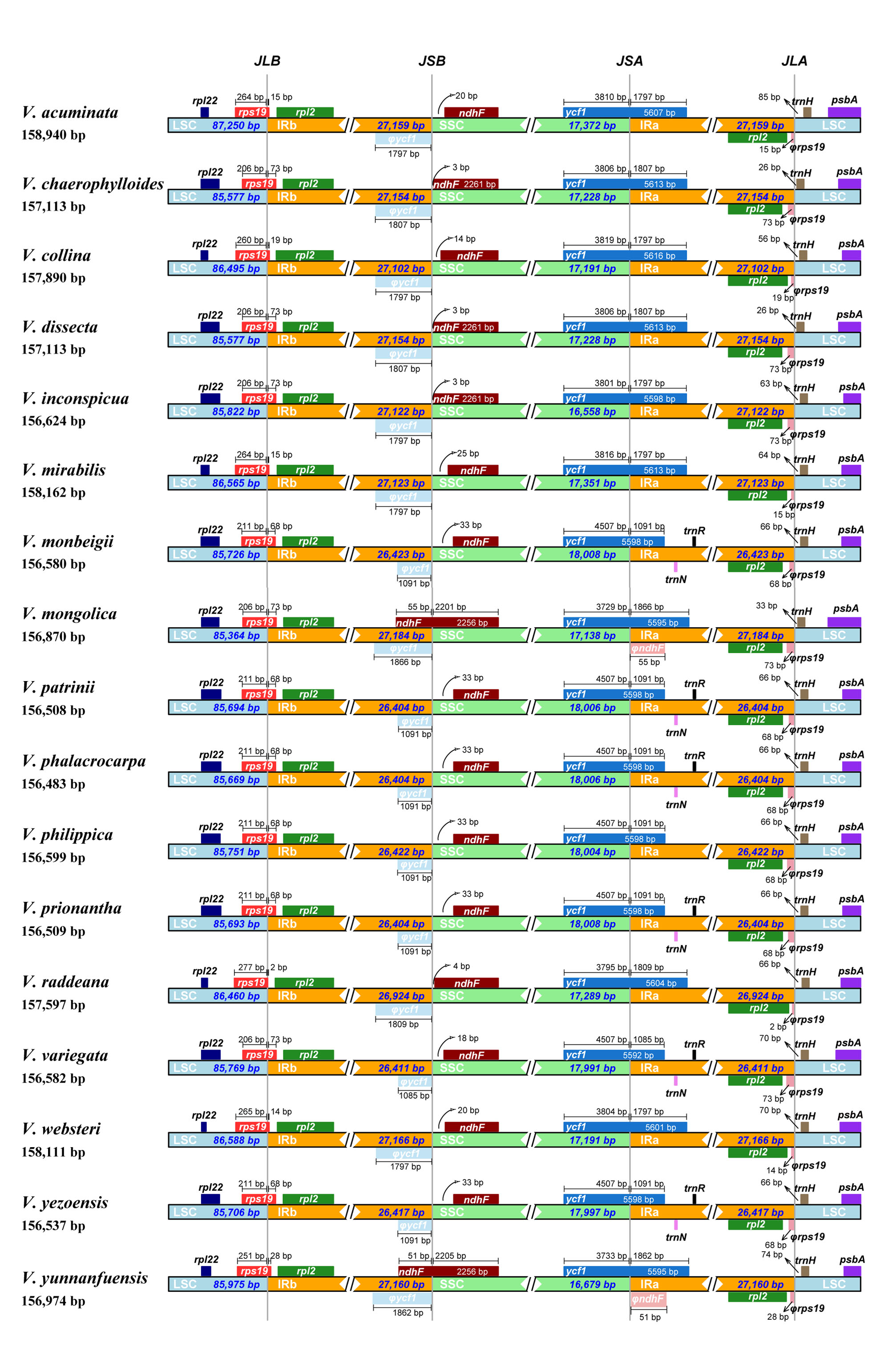

Supplement: Supplementary file 1 — Additional file 1: Fig. S1. Comparisons of LSC, SSC, and IR region borders among 17 chloroplast genomes. Fig. S2. Repeat sequences analysis of 17 cp genomes. Fig. S3. Maximum Likelihood (ML) and Bayesian Inference (BI) phylogenetic trees are based on 16 highly diverged regions. Fig. S4. The variable sites in ndhF, rpl22, and ycf1 of Viola philippica. Fig. S5. Maximum Likelihood (ML) and Bayesian Inference (BI) phylogenetic trees are based on complete chloroplast genome. Fig. S6. Original electrophoretogram for four sequence fragments with unique variable sites in 14 newly sequenced Viola species. [file 12864_2022_8727_MOESM1_ESM.zip › Additional files 1/Figure_S1.jpg]

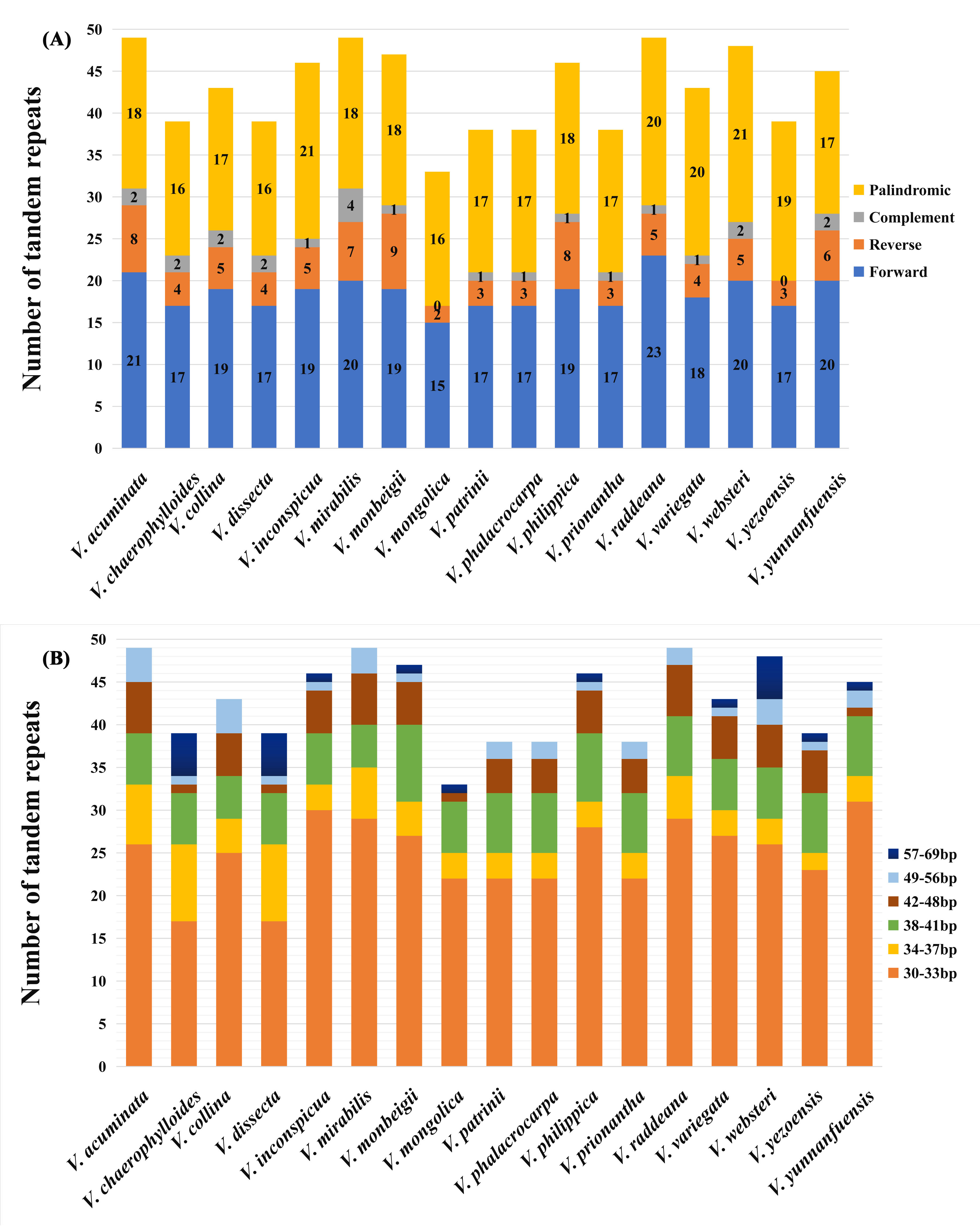

Supplement: Supplementary file 1 — Additional file 1: Fig. S1. Comparisons of LSC, SSC, and IR region borders among 17 chloroplast genomes. Fig. S2. Repeat sequences analysis of 17 cp genomes. Fig. S3. Maximum Likelihood (ML) and Bayesian Inference (BI) phylogenetic trees are based on 16 highly diverged regions. Fig. S4. The variable sites in ndhF, rpl22, and ycf1 of Viola philippica. Fig. S5. Maximum Likelihood (ML) and Bayesian Inference (BI) phylogenetic trees are based on complete chloroplast genome. Fig. S6. Original electrophoretogram for four sequence fragments with unique variable sites in 14 newly sequenced Viola species. [file 12864_2022_8727_MOESM1_ESM.zip › Additional files 1/Figure_S2.jpg]

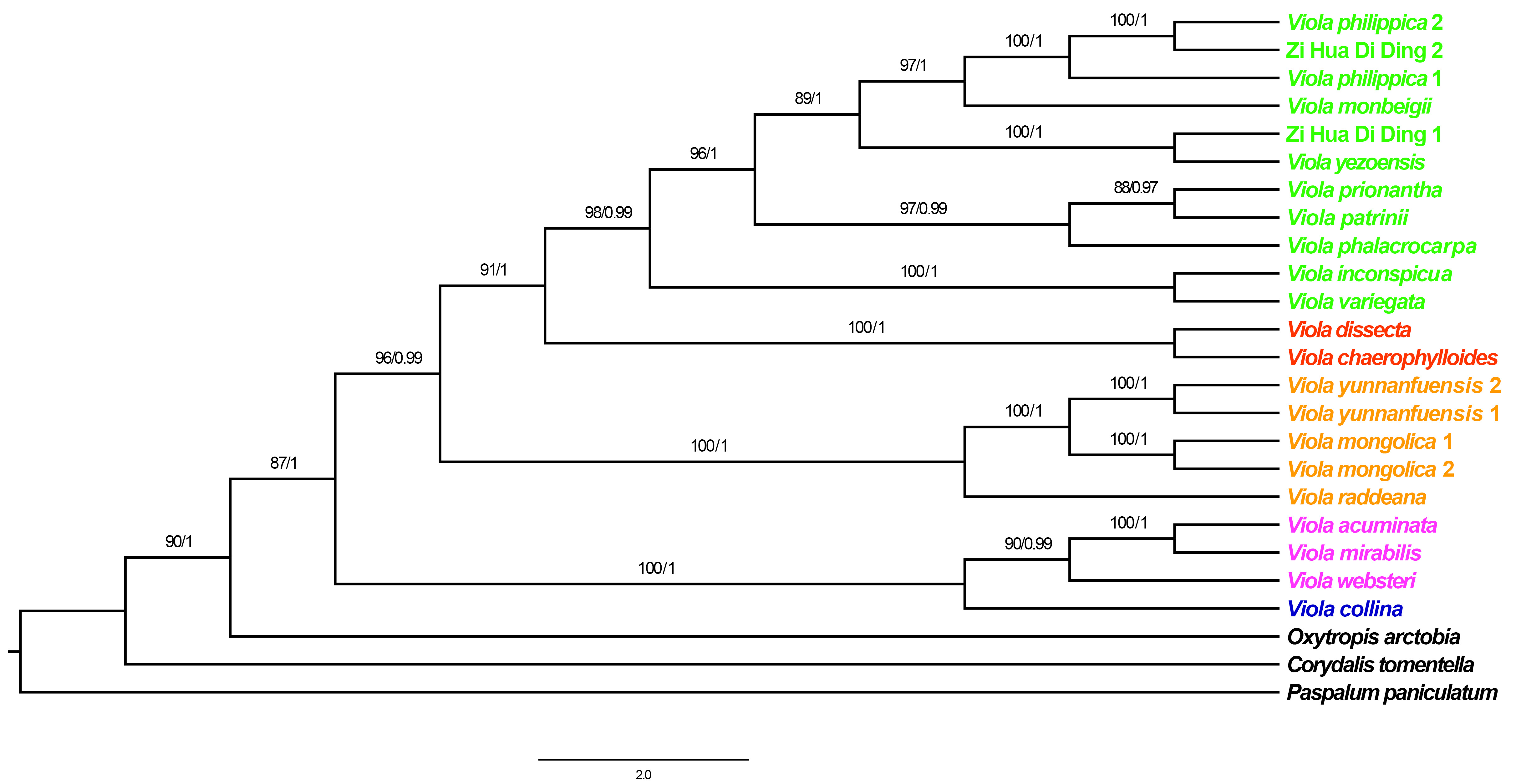

Supplement: Supplementary file 1 — Additional file 1: Fig. S1. Comparisons of LSC, SSC, and IR region borders among 17 chloroplast genomes. Fig. S2. Repeat sequences analysis of 17 cp genomes. Fig. S3. Maximum Likelihood (ML) and Bayesian Inference (BI) phylogenetic trees are based on 16 highly diverged regions. Fig. S4. The variable sites in ndhF, rpl22, and ycf1 of Viola philippica. Fig. S5. Maximum Likelihood (ML) and Bayesian Inference (BI) phylogenetic trees are based on complete chloroplast genome. Fig. S6. Original electrophoretogram for four sequence fragments with unique variable sites in 14 newly sequenced Viola species. [file 12864_2022_8727_MOESM1_ESM.zip › Additional files 1/Figure_S3.jpg]

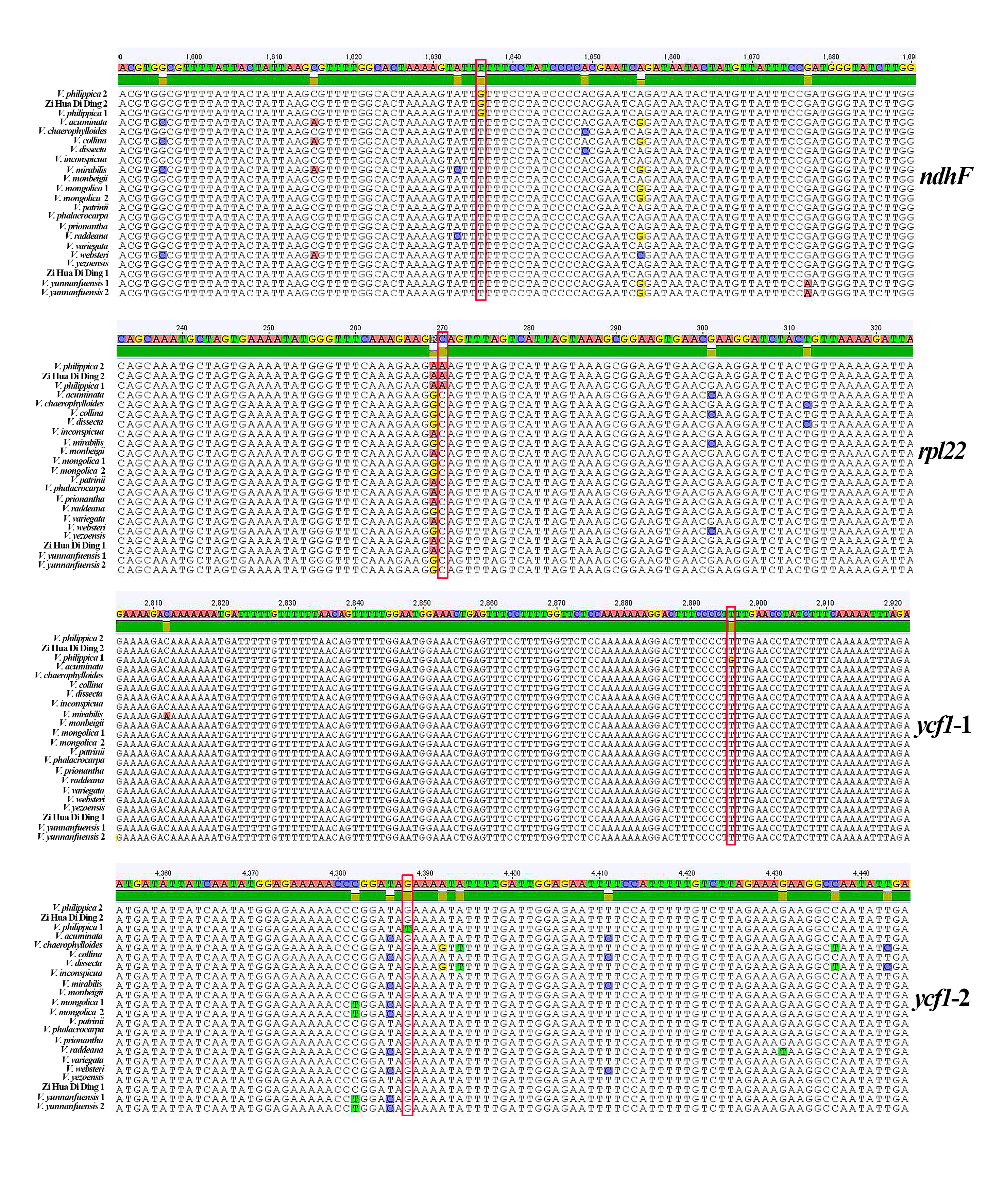

Supplement: Supplementary file 1 — Additional file 1: Fig. S1. Comparisons of LSC, SSC, and IR region borders among 17 chloroplast genomes. Fig. S2. Repeat sequences analysis of 17 cp genomes. Fig. S3. Maximum Likelihood (ML) and Bayesian Inference (BI) phylogenetic trees are based on 16 highly diverged regions. Fig. S4. The variable sites in ndhF, rpl22, and ycf1 of Viola philippica. Fig. S5. Maximum Likelihood (ML) and Bayesian Inference (BI) phylogenetic trees are based on complete chloroplast genome. Fig. S6. Original electrophoretogram for four sequence fragments with unique variable sites in 14 newly sequenced Viola species. [file 12864_2022_8727_MOESM1_ESM.zip › Additional files 1/Figure_S4.jpg]

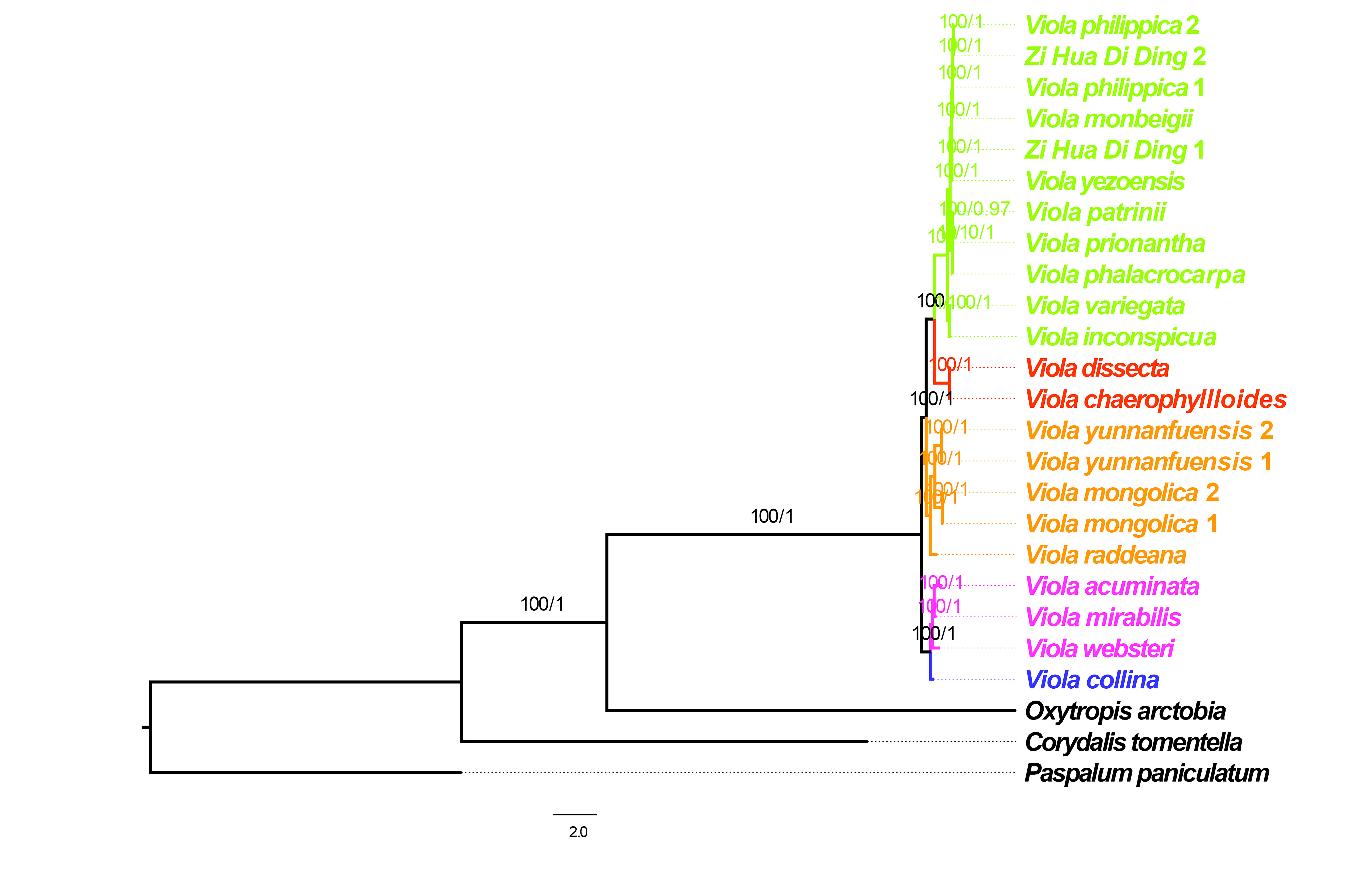

Supplement: Supplementary file 1 — Additional file 1: Fig. S1. Comparisons of LSC, SSC, and IR region borders among 17 chloroplast genomes. Fig. S2. Repeat sequences analysis of 17 cp genomes. Fig. S3. Maximum Likelihood (ML) and Bayesian Inference (BI) phylogenetic trees are based on 16 highly diverged regions. Fig. S4. The variable sites in ndhF, rpl22, and ycf1 of Viola philippica. Fig. S5. Maximum Likelihood (ML) and Bayesian Inference (BI) phylogenetic trees are based on complete chloroplast genome. Fig. S6. Original electrophoretogram for four sequence fragments with unique variable sites in 14 newly sequenced Viola species. [file 12864_2022_8727_MOESM1_ESM.zip › Additional files 1/Figure_S5.jpg]

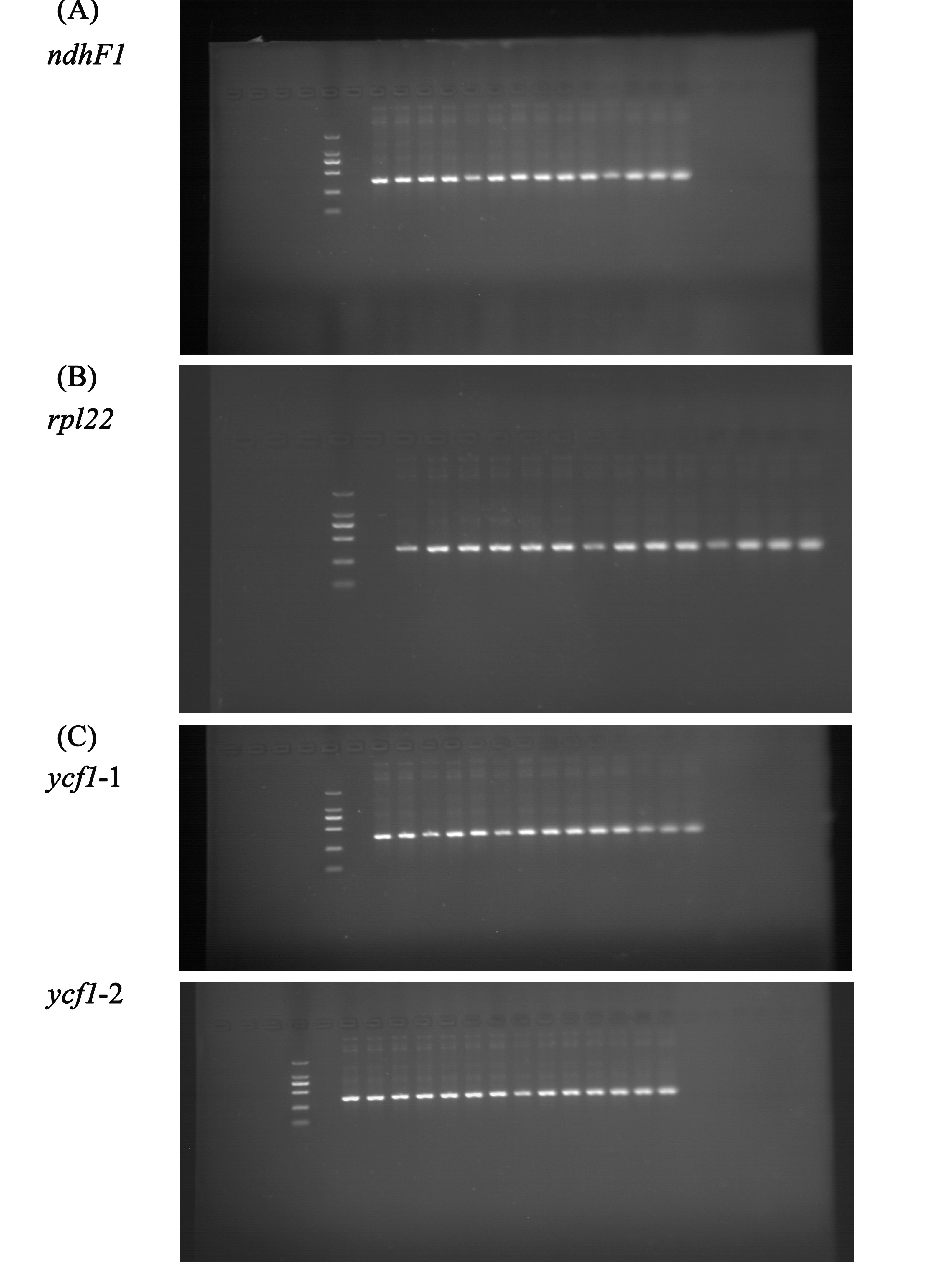

Supplement: Supplementary file 1 — Additional file 1: Fig. S1. Comparisons of LSC, SSC, and IR region borders among 17 chloroplast genomes. Fig. S2. Repeat sequences analysis of 17 cp genomes. Fig. S3. Maximum Likelihood (ML) and Bayesian Inference (BI) phylogenetic trees are based on 16 highly diverged regions. Fig. S4. The variable sites in ndhF, rpl22, and ycf1 of Viola philippica. Fig. S5. Maximum Likelihood (ML) and Bayesian Inference (BI) phylogenetic trees are based on complete chloroplast genome. Fig. S6. Original electrophoretogram for four sequence fragments with unique variable sites in 14 newly sequenced Viola species. [file 12864_2022_8727_MOESM1_ESM.zip › Additional files 1/Figure_S6.jpg]
