# Supplementary material for: Application of chloroplast genome in the identification of Traditional Chinese Medicine Viola philippica
Source: BMC Genomics. 2022 Jul 27;23:540. doi: 10.1186/s12864-022-08727-x (PMC9327190; doi:10.1186/s12864-022-08727-x)
Supplement: Supplementary file 2 — Additional file 2: Table S1. A list of genes found in the cp genomes of 17 Viola species. Table S2. SSR distributed situation in the 17 Viola cp genomes. Table S3. Results of genetic distance analysis. Table S4. Summary of amplified nucleotide sequences and GenBank accession numbers. Table S5. Morphological characteristics for analysis. [file 12864_2022_8727_MOESM2_ESM.docx]

**Table S1** A list of genes found in the plastomes of 17 *Viola* species.

| Category | Group of genes | Name of genes |
| --- | --- | --- |
| Genes for photosynthesis Subunits | ATP synthase | *atpA*^1^, *atpB*^1^, *atpE*^1^, *atpF*^1^, *atpH*^1^, *atpI*^1^ |
|  | NADH-dehydrogenase | *ndhA*^1^, *ndhB*^1^, *ndhC*^1^, *ndhD*^1^, *ndhE*^1^, *ndhF*^1^, *ndhG*^1^, *ndhH*^1^, *ndhI*^1^, *ndhJ*^1^, *ndhK*^1^ |
|  | cytochrome b/f | *petA*^1^, *petB*^1^, *petD*^1^, *petG*^1^, *petL*^1^, *petN*^1^ |
|  | photosystem I | *psaA*^1^, *psaB*^1^, *psaC*^1^, *psaI*^1^, *psaJ*^1^ |
|  | photosystem II | *psbA*^1^, *psbB*^1^, *psbC*^1^, *psbD*^1^, *psbE*^1^, *psbF*^1^, *psbH*^1^, *psbI*^1^, *psbJ*^1^, *psbK*^1^, *psbL*^1^, *psbM*^1^, *psbN*^1^, *psbT*^1^, *psbZ*^1^ |
|  | rubisco | *rbcL*^1^ |
| Self-replication | Large subunit of ribosome | *rpl2*^1^, *rpl14*^1^, *rpl16*^1^, *rpl20*^1^, *rpl21*^1^, *rpl22*^1^, *rpl23*^1^, *rpl33*^1^ |
|  | DNA dependent RNA polymerase | *rpoA*^1^, *rpoB*^1^, *rpoC1*^1^, *rpoC2*^1^ |
|  | Small subunit of ribosome | *rps2*^1^, *rps3*^1^, *rps4*^1^, *rps7*^1^, *rps8*^1^, *rps11*^1^, *rps12*^1^, *rps14*^1^, *rps15*^1^, *rps18*^1^, *rps19*^1^ |
|  | rRNA Genes rrn | *rrn4.5*^1^, *rrn5*^1^, *rrn16*^1^, *rrn23*^1^ |
|  | tRNA Genes trn | *trnA-UGC*^1^, *trnC-GCA*^1^, *trnD-GUC*^1^, *trnE-UUC*^1^, *trnF-GAA*^1^, *trnfM-CAU*^1^, *trnG-UCC*^1^, *trnG-GCC*^1^, *trnH-GUG*^1^, *trnI-GAU*^1^, *trnI-CAU*^1^, *trnK-UUU*^1^, *trnL-CAA*^1^, *trnL-UAG*^1^, *trnL-UAA*^1^, *trnM-CAU*^1^, *trnN-GUU*^1^, *trnP-UGG*^1^, *trnQ-UUG*^1^, *trnR-UCU*^1^, *trnR-ACG*^1^, *trnS-GCU*^1^, *trnS-GGA*^1^, *trnS-UGA*^1^, *trnT-GGU*^1^, *trnT-UGU*^1^, *trnV-GAC*^1^, *trnV-UAC*^1^, *trnW-CCA*^1^, *trnY-GUA*^1^ |
| Other genes | Acetyl-CoA-carboxylase genes | *accD*^1^ |
|  | c-type cytochrom synthesis gene | *ccsA*^1^ |
|  | Envelop membrane protein genes | *cemA*^1^ |
|  | Protease clp genes | *clpP*^2^ |
| Unkown function | Maturase genes | *matK*^1^ |
|  | Conserved open reading | *ycf1*^1^, *ycf2*^1^, *ycf3*^2^, *ycf4*^1^ |

1 and 2 indicate one- and two-intron containing genes, respectively.

**Table S2** SSR distributed situation in the 17 *Viola* plastomes.

| Species | Total SSRs | A/T | | C/G | | AT/TA | | AATT/AATT | LSC | SSC | IRa | IRb |
| --- | --- | --- | --- | --- | --- | --- | --- | --- | --- | --- | --- | --- |
|  |  | **A** | **T** | **C** | **G** | **AT** | **TA** | **ATTA** |  |  |  |  |
| *Viola acuminata* | 34 | 14 | 15 | 2 | - | 1 | 2 | - | 26 | 8 | - | - |
| *V. chaerophylloides* | 26 | 9 | 15 | 2 | - | - | - | - | 18 | 6 | 1 | 1 |
| *V. collina* | 32 | 10 | 17 | 2 | - | 2 | 1 | - | 23 | 7 | 1 | 1 |
| *V. dissecta* | 26 | 9 | 15 | 2 | - | - | - | - | 18 | 6 | 1 | 1 |
| *V. inconspicua* | 27 | 5 | 16 | - | - | 2 | 4 | - | 19 | 6 | 1 | 1 |
| *V. mirabilis* | 34 | 13 | 16 | 1 | - | 2 | 2 | - | 24 | 8 | 1 | 1 |
| *V. monbeigii* | 24 | 7 | 10 | 1 | - | 2 | 4 | - | 12 | 6 | 3 | 3 |
| *V. mongolica* | 19 | 4 | 10 | 1 | 2 | - | 2 | - | 13 | 4 | 1 | 1 |
| *V. patrinii* | 23 | 4 | 9 | 3 | - | 3 | 4 | - | 16 | 5 | 1 | 1 |
| *V. phalacrocarpa* | 25 | 6 | 9 | 3 | - | 3 | 4 | - | 16 | 7 | 1 | 1 |
| *V. philippica* | 22 | 4 | 11 | 1 | - | 2 | 4 | - | 15 | 5 | 1 | 1 |
| *V. prionantha* | 23 | 4 | 9 | 3 | - | 3 | 4 | - | 16 | 5 | 1 | 1 |
| *V. raddeana* | 27 | 5 | 14 | 1 | 1 | 1 | 5 | - | 20 | 7 | - | - |
| *V. variegata* | 27 | 6 | 14 | 1 | - | 2 | 4 | - | 16 | 9 | 1 | 1 |
| *V. websteri* | 33 | 9 | 17 | 1 | 1 | 2 | 2 | 1 | 24 | 7 | 1 | 1 |
| *V. yezoensis* | 25 | 6 | 14 | - | - | 2 | 3 | - | 17 | 4 | 2 | 2 |
| *V. yunnanfuensis* | 23 | 4 | 13 | 1 | 1 | 1 | 3 | - | 18 | 3 | 1 | 1 |

**Table S3** Estimates of evolutionary divergence between chloroplast genome of 17Viola species.

|  | ***V. philippica*** | ***V. acuminata*** | ***V. chaerophylloides*** | ***V. collina*** | ***V. dissecta*** | ***V. inconspicua*** | ***V. mirabilis*** | ***V. monbeigii*** | ***V. mongolica*** | ***V. patrinii*** | ***V. phalacrocarpa*** | ***V. prionantha*** | ***V. raddeana*** | ***V. variegata*** | ***V. websteri*** | ***V. yezoensis*** | ***V. yunnanfuensis*** |
| --- | --- | --- | --- | --- | --- | --- | --- | --- | --- | --- | --- | --- | --- | --- | --- | --- | --- |
| ***V. philippica*** |  |  |  |  |  |  |  |  |  |  |  |  |  |  |  |  |  |
| ***V. acuminata*** | **0.0144661062** |  |  |  |  |  |  |  |  |  |  |  |  |  |  |  |  |
| ***V. chaerophylloides*** | **0.0097461550** | **0.0134018045** |  |  |  |  |  |  |  |  |  |  |  |  |  |  |  |
| ***V. collina*** | **0.0128018683** | **0.0041817695** | **0.0119089217** |  |  |  |  |  |  |  |  |  |  |  |  |  |  |
| ***V. dissecta*** | **0.0097461550** | **0.0134018045** | **0.0000000000** | **0.0119089217** |  |  |  |  |  |  |  |  |  |  |  |  |  |
| ***V. inconspicua*** | **0.0026649329** | **0.0134512161** | **0.0092882585** | **0.0117806006** | **0.0092882585** |  |  |  |  |  |  |  |  |  |  |  |  |
| ***V. mirabilis*** | **0.0131941216** | **0.0030443434** | **0.0123344188** | **0.0027897540** | **0.0123344188** | **0.0122614344** |  |  |  |  |  |  |  |  |  |  |  |
| ***V. monbeigii*** | **0.0004041951** | **0.0144634159** | **0.0097980849** | **0.0126571398** | **0.0097980849** | **0.0026732808** | **0.0133502088** |  |  |  |  |  |  |  |  |  |  |
| ***V. mongolica*** | **0.0122065255** | **0.0116196230** | **0.0114779469** | **0.0096551823** | **0.0114779469** | **0.0111642743** | **0.0102874648** | **0.0122421383** |  |  |  |  |  |  |  |  |  |
| ***V. patrinii*** | **0.0015909430** | **0.0140334572** | **0.0098640554** | **0.0124401434** | **0.0098640554** | **0.0024704933** | **0.0130234021** | **0.0015206964** | **0.0120535618** |  |  |  |  |  |  |  |  |
| ***V. phalacrocarpa*** | **0.0015982952** | **0.0140428149** | **0.0098799624** | **0.0124491724** | **0.0098799624** | **0.0024562130** | **0.0130325994** | **0.0015351376** | **0.0120484526** | **0.0000283461** |  |  |  |  |  |  |  |
| ***V. prionantha*** | **0.0015980454** | **0.0140406062** | **0.0098712292** | **0.0124473012** | **0.0098712292** | **0.0024776335** | **0.0130305500** | **0.0015277916** | **0.0120607494** | **0.0000070853** | **0.0000354321** |  |  |  |  |  |  |
| ***V. raddeana*** | **0.0108175488** | **0.0102817731** | **0.0099384829** | **0.0084563892** | **0.0099384829** | **0.0102829973** | **0.0089722675** | **0.0106864867** | **0.0060065837** | **0.0107636228** | **0.0107653189** | **0.0107707842** |  |  |  |  |  |
| ***V. variegata*** | **0.0023970410** | **0.0138363521** | **0.0091574698** | **0.0122101434** | **0.0091574698** | **0.0012118620** | **0.0125964212** | **0.0025047498** | **0.0116379372** | **0.0024743675** | **0.0024460657** | **0.0024814602** | **0.0102643812** |  |  |  |  |
| ***V. websteri*** | **0.0142180095** | **0.0053473672** | **0.0133780220** | **0.0038745440** | **0.0133780220** | **0.0132631504** | **0.0036741890** | **0.0142211704** | **0.0110695042** | **0.0138780367** | **0.0138875944** | **0.0138922739** | **0.0100081089** | **0.0136609083** |  |  |  |
| ***V. yezoensis*** | **0.0007804740** | **0.0143369176** | **0.0097523520** | **0.0127214251** | **0.0097523520** | **0.0025452570** | **0.0132008953** | **0.0007522212** | **0.0122483620** | **0.0013360053** | **0.0013504197** | **0.0013431118** | **0.0106645261** | **0.0024055227** | **0.0141704177** |  |  |
| ***V. yunnanfuensis*** | **0.0121385992** | **0.0111604593** | **0.0112259546** | **0.0093855498** | **0.0112259546** | **0.0111898760** | **0.0099479055** | **0.0119567568** | **0.0046486680** | **0.0117825100** | **0.0117843783** | **0.0117897164** | **0.0058668189** | **0.0115841527** | **0.0109965537** | **0.0119196989** |  |

**Table S4** Summary of amplified nucleotide sequences and GenBank accession numbers.

| Species | *ndhF* | *rpl22* | *ycf1*-1 | *ycf1*-2 |
| --- | --- | --- | --- | --- |
| *Viola acuminata* | MZ407852 | MZ407866 | MZ407880 | MZ407894 |
| *V. chaerophylloides* | MZ407853 | MZ407867 | MZ407881 | MZ407895 |
| *V. collina* | MZ407854 | MZ407868 | MZ407882 | MZ407896 |
| *V. dissecta* | MZ407855 | MZ407869 | MZ407883 | MZ407897 |
| *V. inconspicua* | MZ407856 | MZ407870 | MZ407884 | MZ407898 |
| *V. monbeigii* | MZ407857 | MZ407871 | MZ407885 | MZ407899 |
| *V. mongolica* | MZ407858 | MZ407872 | MZ407886 | MZ407900 |
| *V. patrinii* | MZ407859 | MZ407873 | MZ407887 | MZ407901 |
| *V. phalacrocarpa* | MZ407860 | MZ407874 | MZ407888 | MZ407902 |
| *V. philippica* | MZ407861 | MZ407875 | MZ407889 | MZ407903 |
| *V. prionantha* | MZ407862 | MZ407876 | MZ407890 | MZ407904 |
| *V. variegata* | MZ407863 | MZ407877 | MZ407891 | MZ407905 |
| *V. yezoensis* | MZ407864 | MZ407878 | MZ407892 | MZ407906 |
| *V. yunnanfuensis* | MZ407865 | MZ407879 | MZ407893 | MZ407907 |

**Table S5** Morphological characteristics for analysis.

| **Species/Character** | **Lobe** | **Length of stipules adnate to petioles** | **Leaf blade base** | **Stigma type** | **Fruit shape** |
| --- | --- | --- | --- | --- | --- |
| *Viola acuminata* | entire leaf | shorter than 1/2 | explanate | immarginate | capsule ellipsoid |
| *V. chaerophylloides* | lobed leaf | shorter than 1/2 | explanate | immarginate | capsule ellipsoid |
| *V. collina* | entire leaf | longer than 1/2 | explanate | immarginate | capsule globose |
| *V. dissecta* | lobed leaf | shorter than 1/2 | explanate | immarginate | capsule ellipsoid |
| *V. inconspicua* | entire leaf | longer than 1/2 | reflexed | margined | capsule ellipsoid |
| *V. mirabilis* | entire leaf | shorter than 1/2 | explanate | immarginate | capsule ellipsoid |
| *V. monbeigii* | entire leaf | longer than 1/2 | reflexed | margined | capsule ellipsoid |
| *V. mongolica* | entire leaf | shorter than 1/2 | explanate | margined | capsule ellipsoid |
| *V. patrinii* | entire leaf | longer than 1/2 | reflexed | margined | capsule ellipsoid |
| *V. phalacrocarpa* | entire leaf | longer than 1/2 | reflexed | margined | capsule ellipsoid |
| *V. philippica* | entire leaf | longer than 1/2 | reflexed | margined | capsule ellipsoid |
| *V. prionantha* | entire leaf | longer than 1/2 | reflexed | margined | capsule ellipsoid |
| *V. raddeana* | entire leaf | shorter than 1/2 | explanate | margined | capsule ellipsoid |
| *V. variegata* | entire leaf | longer than 1/2 | reflexed | margined | capsule ellipsoid |
| *V. websteri* | entire leaf | shorter than 1/2 | explanate | immarginate | capsule ellipsoid |
| *V. yezoensis* | entire leaf | longer than 1/2 | reflexed | margined | capsule ellipsoid |
| *V. yunnanfuensis* | entire leaf | shorter than 1/2 | explanate | margined | capsule ellipsoid |
